# Epidemiology and risk factors of multidrug-resistant tuberculosis in Saudi Arabia: a systematic review and meta-analysis

**DOI:** 10.3389/fpubh.2026.1824576

**Published:** 2026-06-12

**Authors:** Rimas Alanazi, Hareer Alghamdi, Raghad Alansari, Shaden Albassam, Hussam Alghamdi, Haneen Altowairqi, Abdullah Al Alshaykh, Rama Alawi, Sitah Alotaibi, Hashim Tatwani, Ruaa Omer, Abdulaziz AlEissa

**Affiliations:** 1 College of Medicine, King Abdulaziz University, Jeddah, Saudi Arabia; 2 College of Medicine, King Saud bin Abdulaziz University for Health Sciences, Jeddah, Saudi Arabia; 3 College of Medicine, King Saud University, Riyadh, Saudi Arabia; 4 Albaha Health Cluster, Albaha, Saudi Arabia; 5 College of Medicine, Taif University, Taif, Saudi Arabia; 6 College of Medicine, King Saud bin Abdulaziz University for Health Sciences, Riyadh, Saudi Arabia; 7 College of Medicine, University of Jeddah, Jeddah, Saudi Arabia; 8 College of Nursing, Princess Nourah bint Abdulrahman University, Riyadh, Saudi Arabia; 9 University of Science and Technology, Khartoum, Sudan; 10 Department of Family Medicine, King Abdullah bin Abdulaziz University Hospital, Princess Nourah bint Abdulrahman University, Riyadh, Saudi Arabia

**Keywords:** epidemiology, MDR-TB, meta-analysis, multidrug-resistant tuberculosis, risk factors, Saudi Arabia, systematic review, tuberculosis

## Abstract

**Background:**

Tuberculosis (TB), a major global health challenge, has been further complicated by the emergence of multidrug-resistant TB (MDR-TB), defined as resistance to at least isoniazid and rifampicin. In Saudi Arabia, TB management continues to face challenges related to population migration, demographic shifts, and comorbidities such as diabetes. Despite multiple local studies, the overall prevalence and risk factors of MDR-TB are not well established. This study aims to estimate the prevalence of MDR-TB in Saudi Arabia and identify its key risk factors.

**Methods:**

A systematic review and meta-analysis were conducted according to the Preferred Reporting Items for Systematic Reviews and Meta-Analyses (PRISMA) guidelines. Searches were conducted in the following databases: MEDLINE, Scopus, Web of Science, Cochrane Library, and Google Scholar. Studies published in English since 2000, involving adult TB patients tested for isoniazid and rifampicin resistance, were included. Quality was assessed using the Methodological Index for Non-Randomized Studies (MINORS) and Appraisal Tool for Cross-Sectional Studies (AXIS) tools. RStudio (v2024.09.1) was used to perform the analysis with a random-effects model, estimating pooled prevalence and odds ratios (ORs). Heterogeneity was evaluated through *I*^2^, and publication bias was assessed using funnel plots.

**Results:**

Sixteen studies conducted in Saudi Arabia were included, encompassing 4,238 TB cases. The pooled prevalence of isoniazid and rifampicin resistance was 15% [95% confidence interval (CI): 7–28%] and 6% (95% CI: 3–9), respectively. MDR-TB was observed in 8% (95% CI: 4–16) of cases. Previous TB treatment was significantly associated with MDR-TB (OR = 7.34, 95% CI: 2.84–18.98, *p* < 0.001). In unadjusted analyses, male sex, Saudi nationality, smoking, diabetes, and pulmonary TB site were not significantly associated with MDR-TB; however, adjusted pooled analyses suggested significant associations for Saudi nationality and smoking, while diabetes remained non-significant.

**Conclusion:**

MDR-TB remains a significant challenge in Saudi Arabia. Standardized diagnostic protocols, enhanced treatment monitoring, and targeted preventive strategies are essential to curb resistance and improve outcomes.

**Systematic review registration:**

https://www.crd.york.ac.uk/PROSPERO/search, identifier CRD420251078514 (PROSPERO).

## 1 Introduction

Tuberculosis (TB) remains one of the leading infectious causes of morbidity and mortality worldwide and continues to pose a significant global public health challenge. According to the World Health Organization, an estimated 10.6 million new TB cases and approximately 1.6 million TB-related deaths were reported globally in 2021, highlighting the persistent burden of the disease despite decades of control efforts (1). Socioeconomic inequalities, disruptions to healthcare systems, and the emergence of drug-resistant strains have further hindered progress toward TB elimination.

Multidrug-resistant tuberculosis (MDR-TB), defined as resistance to at least isoniazid and rifampicin, which are the two most potent first-line anti-TB drugs, represents a major threat to TB control programs worldwide (2). MDR-TB is associated with prolonged treatment duration, increased toxicity, substantially higher costs, and poorer treatment outcomes compared to drug-susceptible TB (3). Patients with MDR-TB experience higher rates of treatment failure, loss to follow-up, and mortality, placing considerable strain on healthcare systems, particularly in countries with limited resources.

In Saudi Arabia, TB remains an ongoing public health concern despite the availability of structured national TB control programs. Several unique epidemiological factors contribute to the complexity of TB control in the Kingdom, including large-scale population movement related to labor migration and religious mass gatherings, rapidly changing demographic patterns, and a high prevalence of chronic comorbid conditions such as diabetes mellitus (4, 5). Diabetes, in particular, is highly prevalent in Saudi Arabia and has been consistently linked to increased susceptibility to TB infection, delayed sputum conversion, and poorer treatment outcomes, potentially facilitating the emergence of drug resistance.

Although multiple local and regional studies have examined MDR-TB in Saudi Arabia, the reported prevalence and associated risk factors vary considerably across studies. Differences in study design, sample size, geographic coverage, diagnostic methods, and population characteristics have resulted in heterogeneous findings that limit the ability to draw robust national conclusions. Furthermore, while several risk factors, such as previous TB treatment, age, smoking, comorbidities, and regional variation, have been explored individually, there is currently no comprehensive synthesis that integrates these findings to provide an overall national estimate of MDR-TB burden and its key determinants (6).

To address these gaps, this study aims to estimate the pooled prevalence of MDR-TB in Saudi Arabia and to identify and summarize the major associated risk factors, including demographic characteristics, behavioral factors, clinical comorbidities, prior TB treatment history, and regional differences. Providing a consolidated national estimate and risk factor profile may support evidence-based policy planning, strengthen TB control strategies, and guide targeted interventions to reduce the burden of MDR-TB in the Kingdom.

## 2 Materials and methods

### 2.1 Review of the literature

This systematic review and meta-analysis were conducted and reported according to the Preferred Reporting Items for Systematic Reviews and Meta-Analyses (PRISMA) 2020 guidelines (7). Before initiating the review, the protocol was registered in the International Prospective Register of Systematic Reviews (PROSPERO) under registration ID: CRD420251078514 (8).

### 2.2 Search strategy

In June 2025, a systematic search was conducted in the following databases: (1) PubMed, (2) Scopus, (3) Cochrane, (4) Web of Science, and (5) Google Scholar. Google Scholar was used as a supplementary search source, and the first 200 results sorted by relevance were screened. The full electronic search strategy is provided in the Supplementary material.

In this study, the primary outcomes of interest were the prevalence of MDR-TB in Saudi Arabia and resistance to individual anti-TB drugs in Saudi Arabia. Secondary outcomes included identified risk factors associated with MDR-TB, expressed as pooled odds ratios (ORs). For risk-factor analyses, the outcome of interest was MDR-TB, defined as resistance to at least both isoniazid and rifampicin, compared with non-MDR-TB, where the included studies provided sufficient stratified data. To avoid conceptual overlap, mono-resistant or non-specific MDR-TB outcomes were not interpreted as MDR-TB unless the original study specifically reported MDR-TB-stratified estimates. Ethical approval was not required because this study was based exclusively on published, anonymized data.

### 2.3 Eligibility criteria

Studies were eligible for inclusion if they were published in English, included adult TB patients (≥ 18 years) or clinical *Mycobacterium tuberculosis* isolates from Saudi Arabia; performed drug-resistance testing for at least both isoniazid and rifampicin; reported extractable data on MDR-TB prevalence and/or stratified data comparing MDR-TB and non-MDR-TB cases; provided statistical results on prevalence or risk factors; were conducted on or after 1 January 2000; and were quantitative observational studies (cross-sectional, case–control, or cohort).

Broad-based studies (e.g., molecular resistance patterns, mutation profiling, second-line resistance, mortality, or co-infection) were included when they contained extractable data on MDR-TB prevalence, first-line drug resistance, or risk factors associated with MDR-TB. Studies that failed to meet these criteria were excluded.

### 2.4 Process of screening

**Figure 1:**
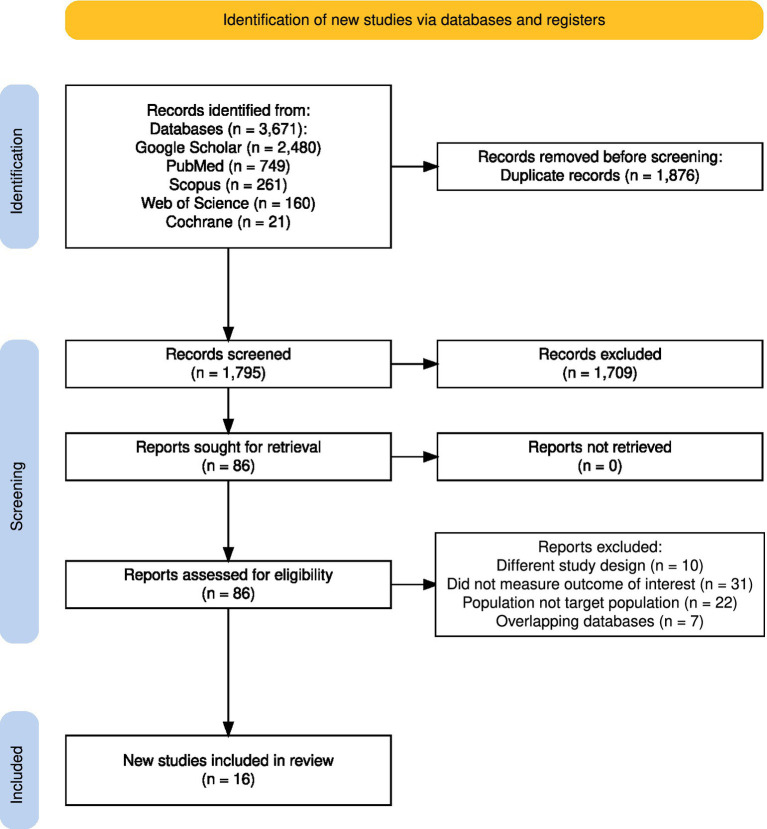
PRISMA flow diagram of the included studies.

Five independent reviewers screened the identified papers’ titles and abstracts using the Rayyan web and mobile app for systematic reviews (9). Duplicate records were identified and removed using Rayyan’s deduplication feature. Any disagreements were resolved through discussion among the reviewers. Formal inter-rater agreement statistics (e.g., kappa) were not calculated. The study selection process is illustrated in the PRISMA flow diagram (Figure 1).

### 2.5 Data extraction

Data were extracted using a standardized Microsoft Excel form and organized into four main domains. Study characteristics included article identification details, publication year, journal, study design, country of origin, sample size, and group distribution where applicable. Participant demographics comprised age (range and mean), sex distribution, smoking status, comorbidities, and other relevant demographic characteristics. Intervention details included the type of intervention, the comparison group, the treatment duration, and the follow-up period. Outcome measures included the prevalence of MDR-TB, resistance patterns to anti-TB drugs, and associated risk factors such as smoking, TB history, comorbidities, and age.

In studies with broader primary aims, only data pertinent to this study were extracted, such as MDR-TB prevalence, isoniazid/rifampicin resistance, first-line drug-resistance trends, and MDR-TB-related risk factors. Unrelated mutation, lineage, second-line resistance, mortality, or co-infection results were not pooled.

### 2.6 Quality assessment

Given the heterogeneity of the included studies, two validation tools were used for quality assessment. Cohort and retrospective studies were evaluated using the Methodological Index for Non-Randomized Studies (MINORS), while cross-sectional studies were assessed using the Appraisal Tool for Cross-Sectional Studies (AXIS). The Newcastle–Ottawa Scale was initially planned for use; however, after piloting, MINORS and AXIS proved more appropriate. This change was recorded as a protocol amendment in PROSPERO. Two reviewers independently assessed each study, and discrepancies were resolved through discussion or consensus.

### 2.7 Statistical analysis and heterogeneity

An R Markdown document (v2.29) in RStudio (v2024.09.1) with R (v4.4.2) was used to perform the meta-analysis of the selected studies. The prevalence with 95% confidence intervals (CIs) was used for the discontinuous outcomes. Additionally, pooled ORs with 95% CIs were used to assess associated risk factors. A random-effects model and the inverse-variance method were used to reduce heterogeneity. The “dmetar” package was used for leave-one-out meta-analysis and related plots to explore heterogeneity further.

The *I*^2^ statistic was interpreted as follows: 0–40% low heterogeneity; 30–60% moderate heterogeneity; and 50–90% substantial heterogeneity, according to the Cochrane Handbook, Chapter 9. Publication bias was assessed using funnel plots.

Subgroup analysis and meta-regression were not performed because the number of studies contributing to most outcomes and risk-factor analyses was limited, and key subgroup variables such as region, diagnostic method, nationality, prior treatment status, and comorbidities were inconsistently reported across studies. Therefore, potential sources of heterogeneity were explored narratively rather than statistically.

## 3 Results

### 3.1 Screening of included studies

The systematic search identified 3,671 records across databases and registers, including Google Scholar (*n* = 2,480), PubMed (*n* = 749), Scopus (*n* = 261), Web of Science (*n* = 160), and the Cochrane Library (*n* = 21). After excluding 1,876 duplicates, 1,795 records were screened based on titles and abstracts, resulting in the exclusion of 1,709 articles. Subsequently, 86 full-text articles were assessed for eligibility, after which 70 articles were excluded based on pre-specified criteria. Finally, 16 studies were included in the systematic review after the inclusion criteria were met (Figure 1).

### 3.2 Characteristics of the studies and quality assessment

Sixteen articles discussing the prevalence of anti-TB drug resistance and associated risk factors were included in this study. The included studies comprised cross-sectional, cohort, and case–control studies. All included studies were conducted in Saudi Arabia. The summary characteristics of the included studies are presented in Table 1. The baseline features of the included studies are outlined in Table 2.

**Table 1:** Study characteristics of the included studies.

Study	Journal	Study design	Publication year	Country	Sample size	MDR-TB-relevant outcomes
Abdelfattah et al. (20)	The Egyptian Journal of Chest Diseases and Tuberculosis	Cross-sectional study	2023	Saudi Arabia	114	MDR-TB prevalence, first-line drug-resistance pattern, comorbidities, and marital status
Abulkalam et al. (21)	Journal of Advances in Medicine and Medical Research	Retrospective cohort study	2021	Saudi Arabia	695	MDR-TB, polyresistant TB, mono-resistant TB, drug-resistance patterns, and diabetes-related risk data
Sambas et al. (22)	Infection and Drug Resistance	Cross-sectional study	2020	Saudi Arabia	158 confirmed TB patients	MDR-TB prevalence, first-line drug-resistance pattern, and risk-factor data
Al-Shahrani et al. (23)	Saudi Medical Journal	Descriptive retrospective cross-sectional study	2021	Saudi Arabia	901	Prevalence of anti-TB drug resistance and associated risk factors
Ahmed-Abakur and Saad Alnour (24)	International Journal of Mycobacteriology	Descriptive cross-sectional study	2019	Saudi Arabia	661	INH resistance, RIF resistance, MDR-TB frequency; mutation data were not pooled
Al-Hayani et al. (25)	Cureus	Retrospective cross-sectional study	2021	Saudi Arabia	472	MDR-TB prevalence, mono-resistant/poly-resistant TB patterns, and sociodemographic/clinical risk factors for MDR-TB
Varghese and Al-Hajoj (26)	The American Journal of Tropical Medicine and Hygiene	Retrospective analysis	2017	Saudi Arabia	2,956 isolates screened; 83 MDR-TB cases analyzed	MDR-TB case count; second-line resistance and mutation data were described but not pooled
Al Ammari et al. (6)	Antimicrobial Resistance and Infection Control	Retrospective cohort study	2018	Saudi Arabia	2,098 patients with positive TB cultures	MDR-TB prevalence and risk factors, rifampicin-resistant TB, and first-line monoresistance patterns
Varghese et al. (13)	PLOS ONE	Cross-sectional genotyping and drug-susceptibility surveillance study	2013	Saudi Arabia	322 MDR-TB isolates	MDR-TB isolate data and MDR-related epidemiological information; lineage data were not pooled
Varghese et al. (27)	Infection, Genetics and Evolution	Cross-sectional molecular study	2012	Saudi Arabia	151	INH resistance, RIF resistance, and MDR-TB classification; mutation/lineage data were not pooled
Varghese et al. (10)	Microbial Drug Resistance	Cross-sectional molecular epidemiological study	2014	Saudi Arabia	415	INH resistance, RIF resistance, and MDR-TB/monoresistance classification; mutation and phylogenetic data were not pooled
Asaad et al. (28)	Journal of Infection and Public Health	Observational cross-sectional study	2012	Saudi Arabia	80 total; 68 confirmed cases analyzed	First-line drug-resistance pattern and MDR-TB prevalence
El Mahalli and Al-Qahtani (14)	Journal of the Egyptian Public Health Association	Case–control observational study	2015	Saudi Arabia	181	Demographic and clinical risk-factor data relevant to drug-resistant TB/MDR-TB synthesis
Samman et al. (29)	Clinical Microbiology and Infectious Disease	Retrospective observational study	2003	Saudi Arabia	147	MDR-TB prevalence, treatment outcome, and treatment-compliance data
Ali Chaudhry et al. (11)	Journal of Epidemiology and Global Health	Retrospective laboratory-based study	2011	Saudi Arabia	1,681	First-line anti-TB drug-resistance rates
Mokhtar et al. (30)	International Journal of General Medicine	Retrospective observational study	2025	Saudi Arabia	131	MDR-TB/XDR-TB frequency and comorbidity-related risk data; mortality outcomes were not pooled

INH, isoniazid; RIF, rifampicin; MDR-TB, multidrug-resistant tuberculosis; XDR-TB, extensively drug-resistant tuberculosis. Some studies had broader primary objectives, such as molecular profiling, second-line resistance, mortality, or co-infection; only extractable data relevant to MDR-TB prevalence, first-line drug resistance, or MDR-TB-associated risk factors were included in the synthesis.

**Table 2:** Study characteristics of the included studies.

Study	Age, years	Male, *n* (%)	Female, *n* (%)	Smoking status	Comorbidities	Follow-up duration
Abdelfattah et al. (20)	Range: 16–91; mean ± SD: 35.5 ± 16.8	81 (71.1%)	33 (28.9%)	NR	62 patients with comorbidities	NR
Abulkalam et al. (21)	>17 years; mean ± SD for DR-TB group: 43.19 ± 16.71	389 (56.0%)	306 (44.0%)	13 smokers; 58 non-smokers; 21 unknown	Hypertension: 16; cardiac disease: 8; HIV: 3; dyslipidemia: 3; malignancy: 1; other comorbidities: 22	Jan 2012–Jan 2021, approximately 108 months
Sambas et al. (22)	Mean ± SD: 43.4 ± 18.7	105 (66.5%)	53 (33.5%)	42 smokers	64 patients with comorbidities	NR
Alshahrani et al. (12)	Range: 0 to >80	503 (55.8%)	398 (44.2%)	29 smokers	Renal disease: 18; liver disease: 26; hyperbilirubinemia: 24; diabetes: 50; HIV: 1	2000–2018, approximately 216 months
Ahmed-Abakur and Saad Alnour (24)	Range: <1 to 80; mean: 34.95	62.2%	37.8%	NR; male predominance in smoking described narratively	NR	NR
Al-Hayani et al. (25)	Mean ± SD: 35 ± 17.1	296 (62.7%)	176 (37.3%)	NR	Diabetes mellitus: 40 (8.5%); renal failure: 11 (2.3%); AIDS: 14 (3.0%); immunosuppression: 9 (1.9%); cancer: 7 (1.5%)	NR
Varghese and Al-Hajoj (26)	Median approximately 40	Approximately 57 (69.2%)	Approximately 26 (30.8%)	NR	NR	NR
Al Ammari et al. (6)	Mean ± SD: 36.6 ± 15.9	1,449 (69.1%)	649 (30.9%)	NR	Diabetes: 176; HIV: 45; lung disease: 4; chronic renal failure: 3; immunosuppression: 1	NR
Varghese et al. (13)	NR	NR	NR	NR	NR	NR
Varghese et al. (27)	Age categories: 16–29, 30–44, 45–59, ≥60	103 (68.2%)	48 (31.8%)	NR	NR	NR
Varghese et al. (10)	NR	NR	NR	NR	NR	NR
Asaad et al. (28)	NR	NR	NR	NR	NR	NR
El Mahalli and Al-Qahtani (14)	Cases: 33.9 ± 7.98; controls: 36.2 ± 10.3	Cases: 55 (68.8%); controls: 83 (82.2%)	Cases: 25 (31.3%); controls: 18 (17.8%)	Cases: 47 (58.8%); controls: 39 (38.6%)	Diabetes: 51 cases (63.8%) vs. 50 controls (49.5%); HIV/AIDS: rare, one patient per group	NR
Samman et al. (29)	NR	75 (51.0%)	72 (49.0%)	NR	NR	6-month standard treatment protocol
Ali Chaudhry et al. (11)	NR	999 (59.4%)	682 (40.6%)	NR	NR	NR
Mokhtar et al. (30)	NR	NR	NR	NR	Renal failure, HIV, autoimmune disease, cancer, diabetes, and respiratory conditions reported	Mortality risk-factor follow-up reported; exact duration NR

NR, not reported; DR-TB, drug-resistant tuberculosis; HIV, human immunodeficiency virus. Values are presented as *n* (%) unless otherwise stated. Age was reported according to the format available in each original study, including mean ± SD, median, range, or age categories.

MINORS scores ranged from 9 to 13, indicating overall moderate methodological quality. The majority of the studies had well-defined objectives and relevant drug-resistance outcomes. The key shortcomings included retrospective data collection, lack of clarity regarding consecutive inclusion in certain studies, lack of reporting on unbiased assessment, and absence of formal sample size calculations (Table 3).

**Table 3:** MINORS quality assessment of included studies.

Study	Aim	Consecutive	Prospective data	Appropriate endpoints	Unbiased assessment	Adequate follow-up	Loss to follow-u*p* <5%	Sample size calculations	Total score
Abulkalam et al. (21)	2	1	0	2	1	2	2	0	10
Samman et al. (29)	2	1	0	2	1	2	1	0	9
Al-Shahrani et al. (23)	2	2	0	2	2	2	2	0	12
Al-Hayani et al. (25)	2	2	0	2	2	2	2	0	12
Varghese and Al-Hajoj (26)	2	1	0	2	2	1	2	0	10
Al Ammari et al. (6)	2	2	1	2	2	2	2	0	13
El Mahalli and Al-Qahtani (14)	2	1	0	2	1	2	2	0	10
Ali Chaudhry et al. (11)	2	2	1	2	2	2	2	0	13
Mokhtar et al. (30)	2	2	0	2	1	1	1	0	9

Each item was scored as 0 = not reported, 1 = reported but inadequate or unclear, and 2 = adequately reported. For cross-sectional and laboratory surveillance studies, “adequate follow-up” was interpreted as adequacy and completeness of the sampling/observation period for the reported resistance outcomes.

AXIS scores ranged from 12 to 17 out of 20, indicating generally moderate-to-good reporting quality among the cross-sectional studies. Most studies had clearly stated aims, appropriate cross-sectional designs, relevant drug-resistance endpoints, and valid laboratory-based outcome assessments (Table 4). Therefore, the evidence included was appropriate for synthesis but should be interpreted with caution.

**Table 4:** AXIS quality assessment of cross-sectional studies.

Study	Clear aim	Appropriate design	Sample size justified	Target population defined	Appropriate sample frame	Representative selection	Non-response addressed	Variables appropriate	Valid measurement methods	Statistical methods clear	Methods sufficiently described	Basic data described	Non-response bias low/unclear	Non-responders described	Results internally consistent	All planned results reported	Conclusions justified	Limitations discussed	Funding/COI reported	Ethics approval reported	Total /20
Abdelfattah et al. (20)	1	1	0	1	1	0	0	1	1	1	1	1	1	0	1	1	1	0	0	0	**13**
Sambas et al. (22)	1	1	0	1	1	1	0	1	1	1	1	1	1	0	1	1	1	1	1	1	**17**
Ahmed-Abakur and Saad Alnour (24)	1	1	0	1	1	0	0	1	1	0	1	1	1	0	1	1	1	0	0	0	**12**
Varghese et al. (13)	1	1	0	1	1	1	0	1	1	1	1	1	1	0	1	1	1	1	1	1	**17**
Varghese et al. (27)	1	1	0	1	1	1	0	1	1	1	1	1	1	0	1	1	1	0	0	0	**14**
Varghese et al. (10)	1	1	0	1	1	1	0	1	1	1	1	1	1	0	1	1	1	0	1	0	**15**
Asaad et al. (28)	1	1	0	1	1	1	0	1	1	1	1	1	1	0	1	1	1	0	0	0	**14**

Each item was scored as 1 = yes/adequately reported and 0 = no, unclear, or not reported.

### 3.3 Primary outcomes

#### 3.3.1 Isoniazid resistance

**Figure 2:**
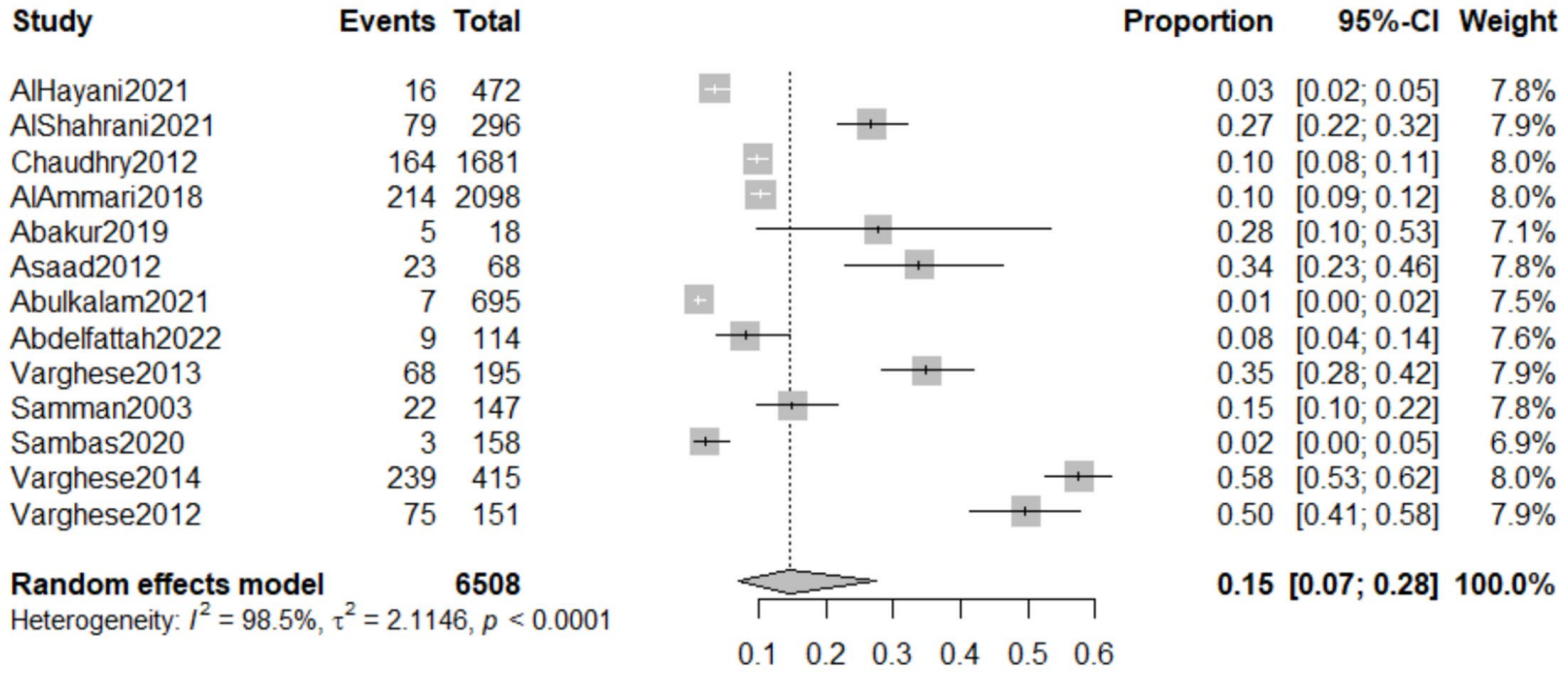
Forest plot of the isoniazid resistance outcome.

**Figure 3:**
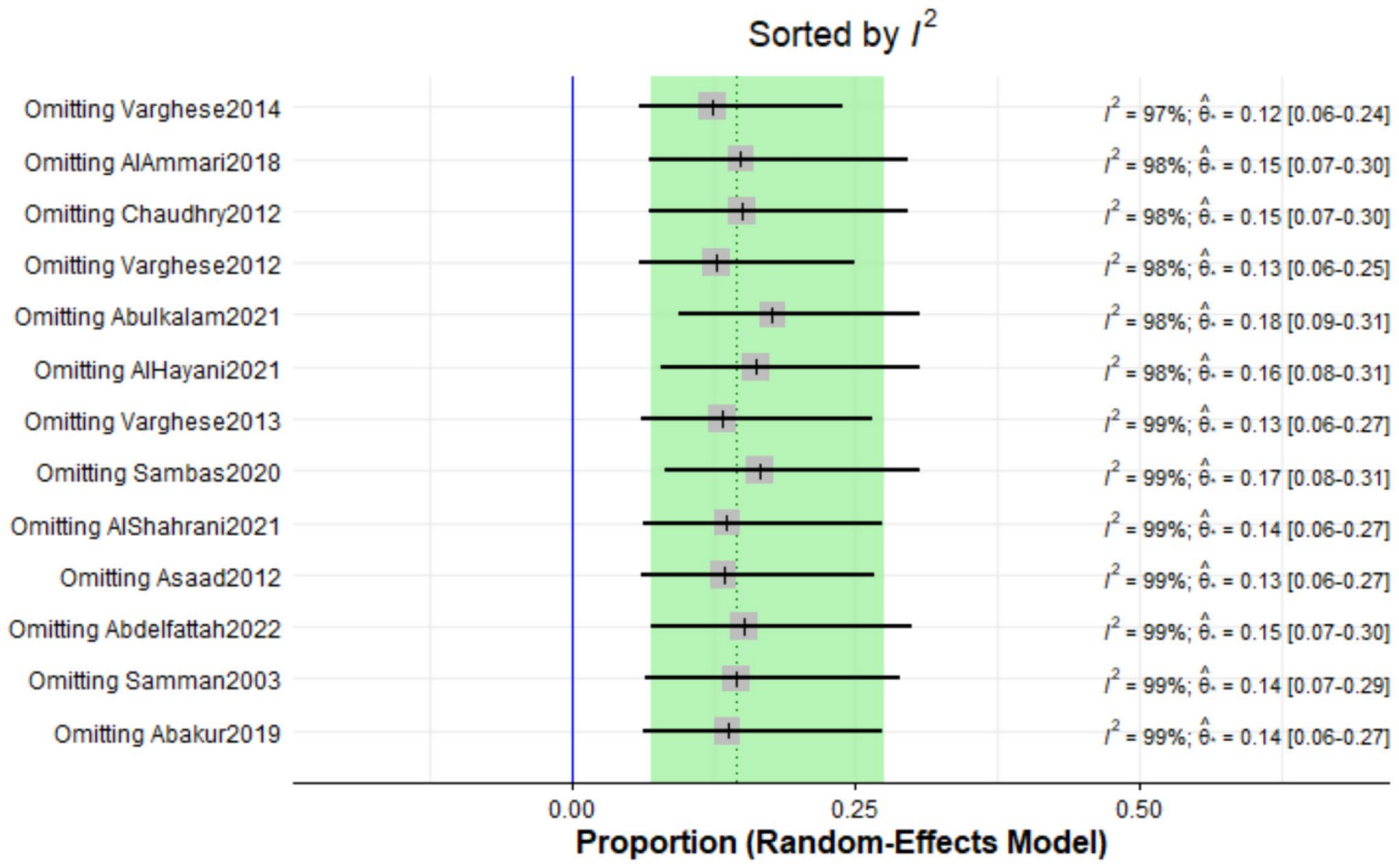
Sensitivity analysis of the isoniazid-resistance outcome.

**Figure 4:**
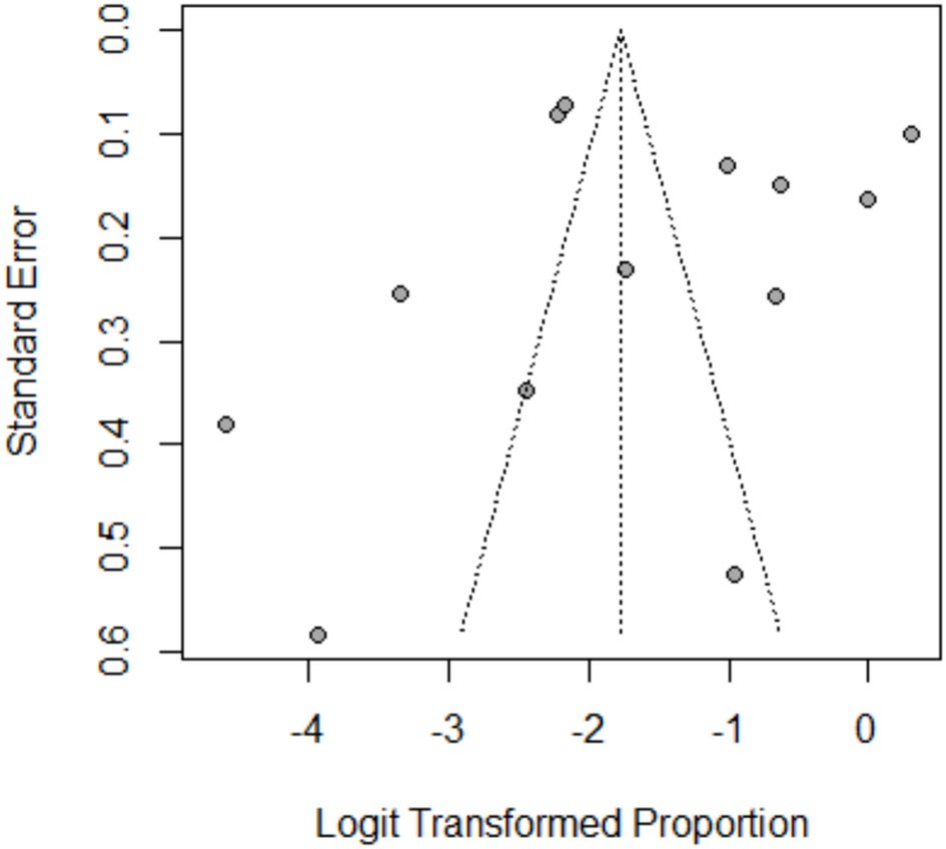
Funnel plot of the isoniazid-resistance outcome.

The pooled prevalence of isoniazid resistance was 0.15 with a 95% CI ranging from 0.07 to 0.28 (Figure 2). Sensitivity analysis revealed substantial heterogeneity among the included studies (*I*^2^ = 98.5%, *p* < 0.0001). The leave-one-out analysis did not resolve the heterogeneity after omitting Varghese et al. (10) (*I*^2^ = 97%) (Figure 3). The funnel plot for the isoniazid-resistance outcome is shown in Figure 4.

#### 3.3.2 Ethambutol resistance

**Figure 5:**
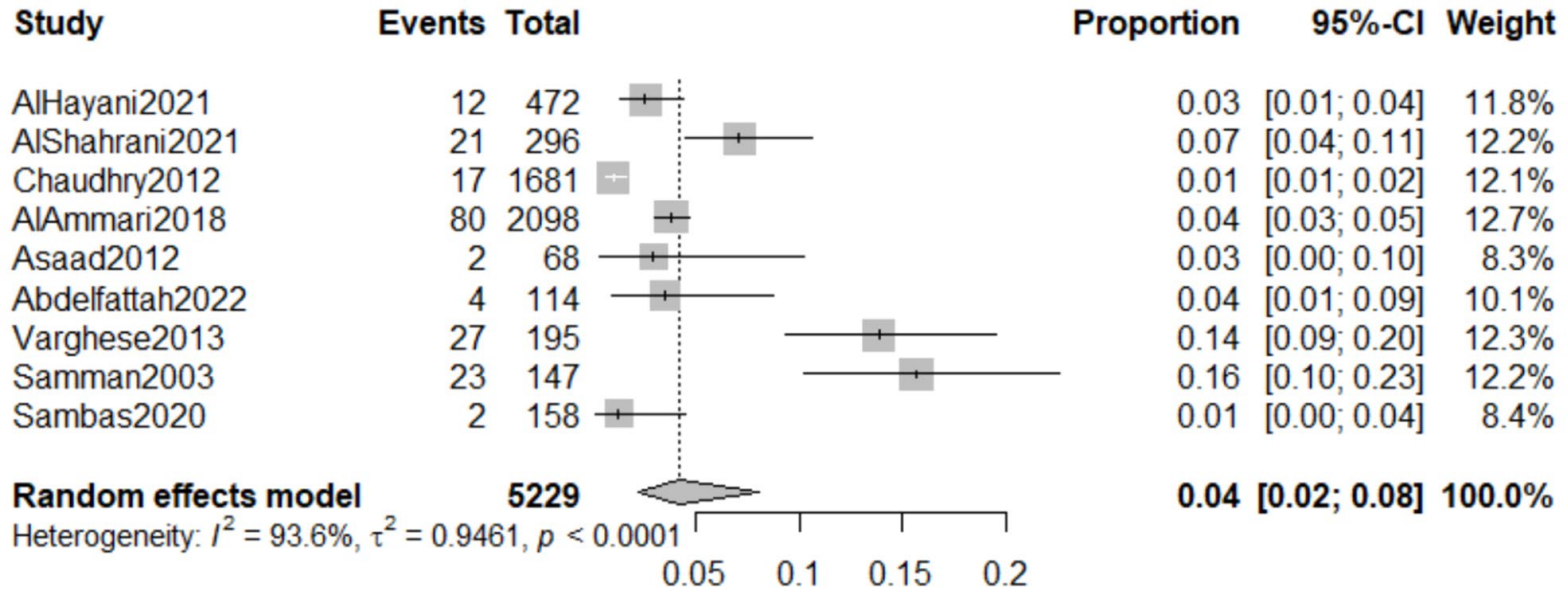
Forest plot of the ethambutol-resistance outcome.

**Figure 6:**
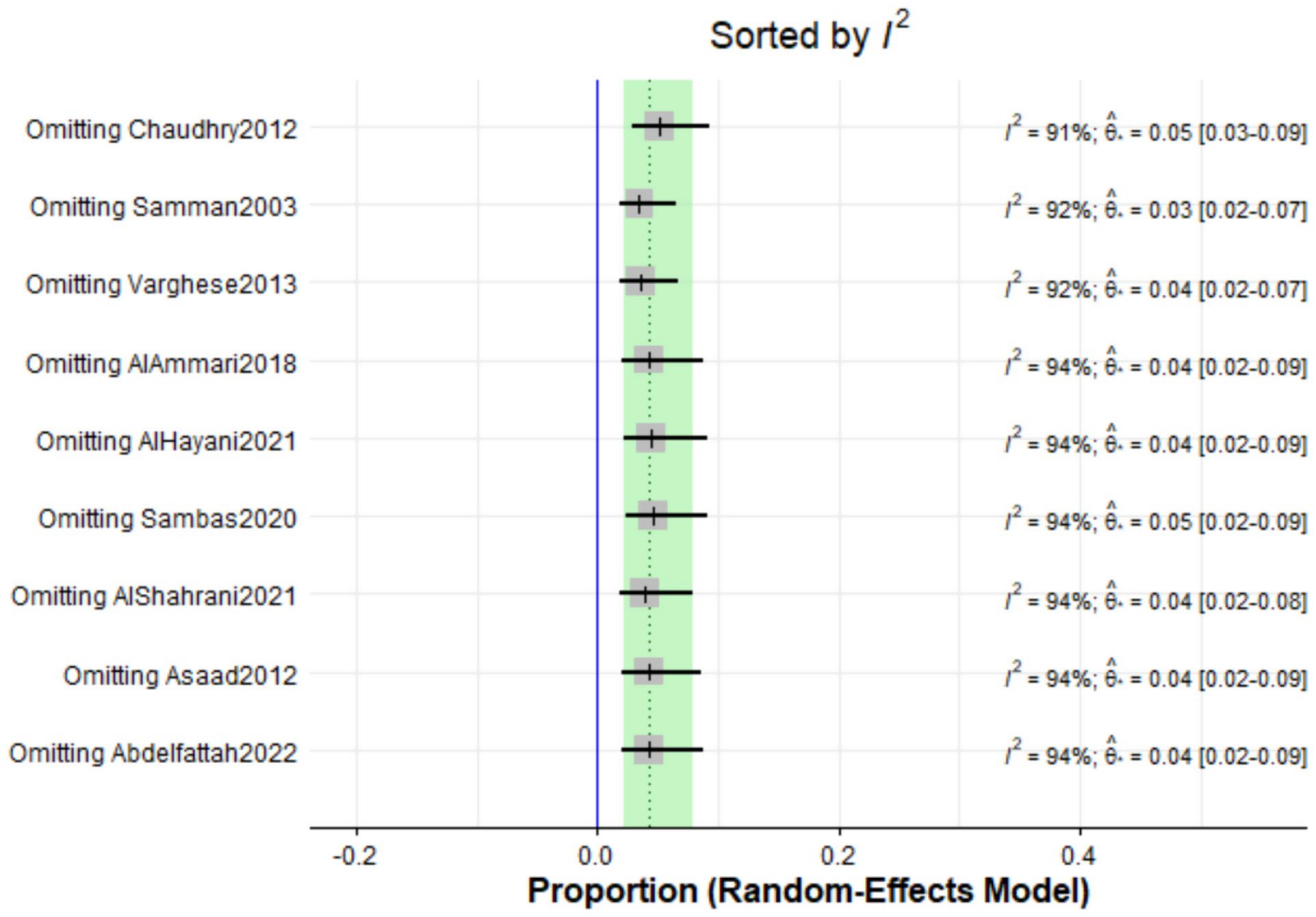
Sensitivity analysis of the ethambutol-resistance outcome.

**Figure 7:**
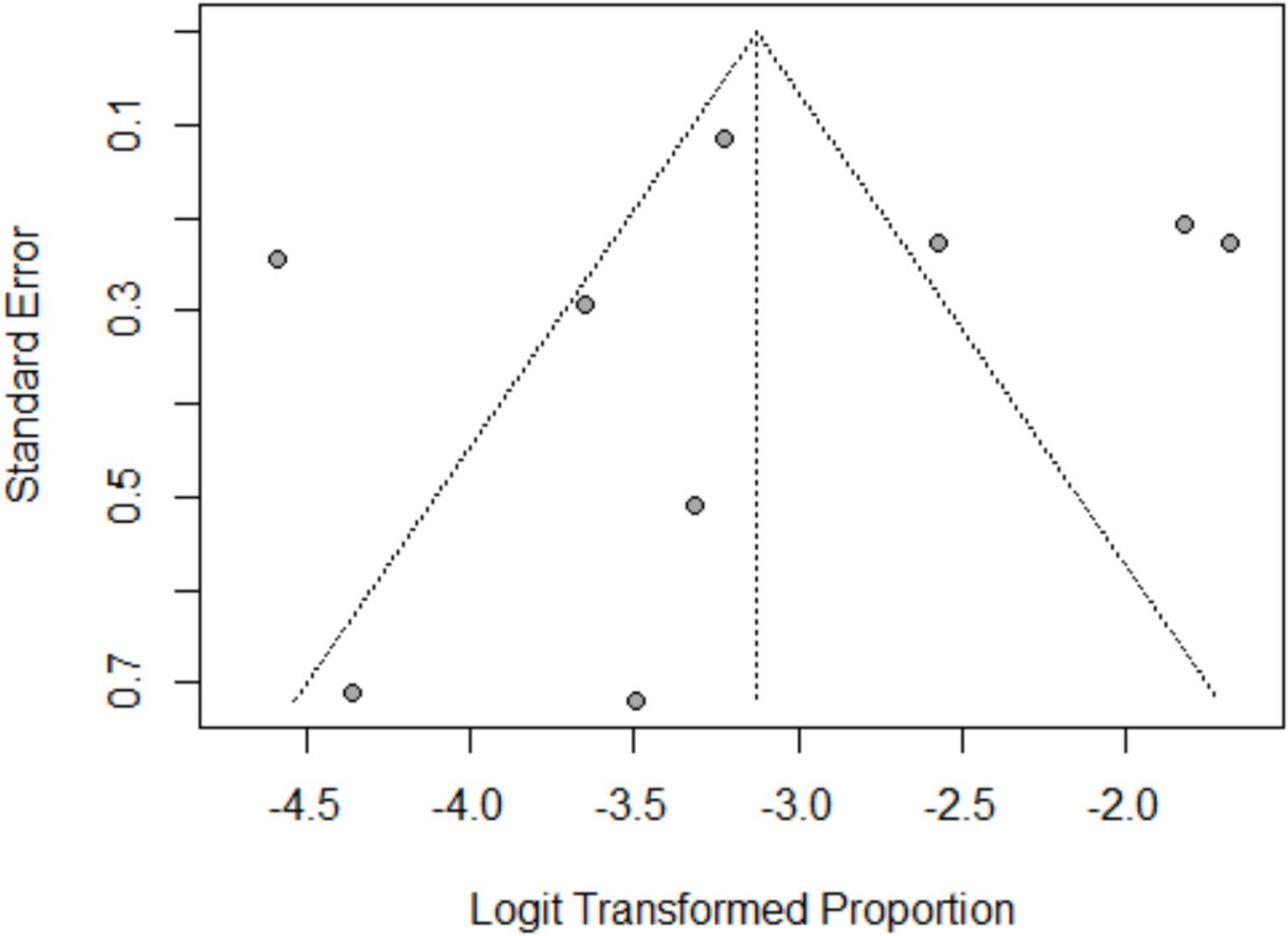
Funnel plot of the ethambutol-resistance outcome.

The pooled prevalence of ethambutol resistance was 0.04 with a 95% CI ranging from 0.02 to 0.08 (Figure 5). Sensitivity analysis revealed substantial heterogeneity among the included studies (*I*^2^ = 93.6%, *p* < 0.0001). The leave-one-out analysis did not resolve the heterogeneity after omitting Ali Chaudhry et al. (11) (*I*^2^ = 91%) (Figure 6). The funnel plot for the ethambutol-resistance outcome is shown in Figure 7.

#### 3.3.3 Pyrazinamide resistance

**Figure 8:**
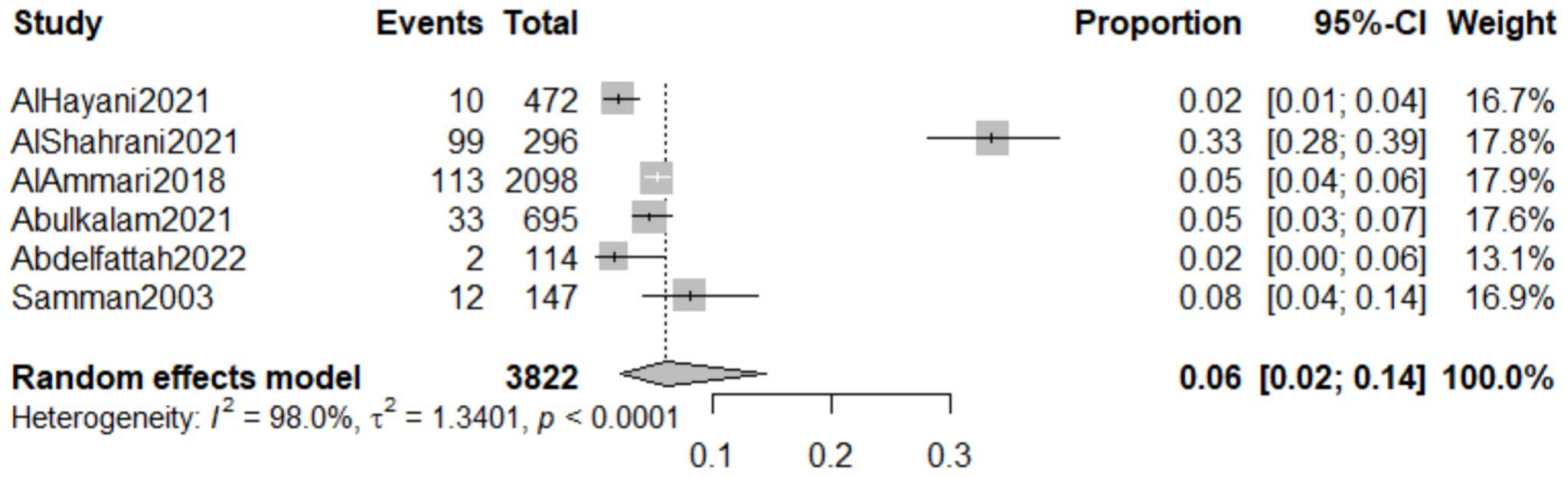
Forest plot of the pyrazinamide-resistance outcome.

**Figure 9:**
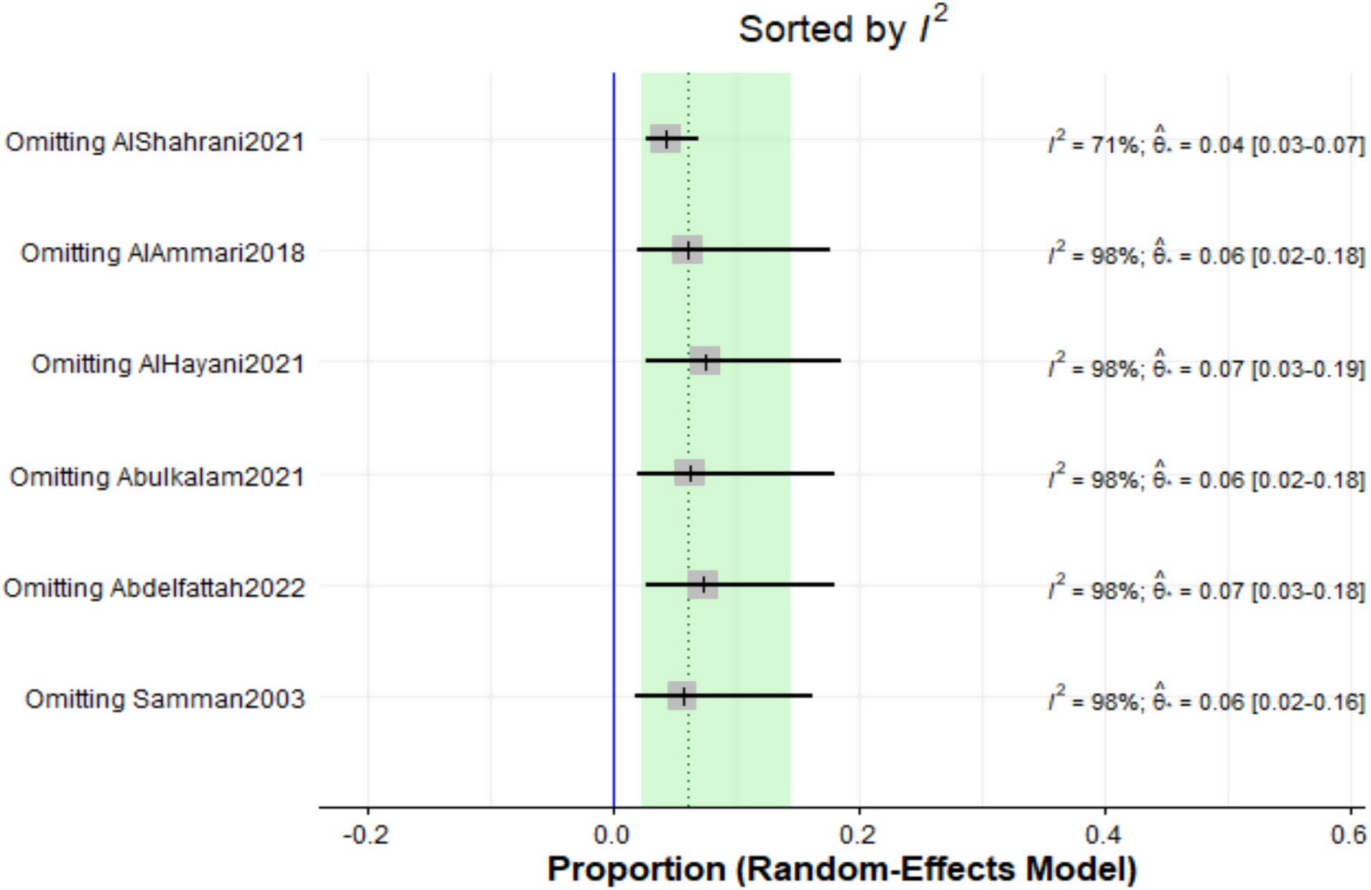
Sensitivity analysis of the pyrazinamide-resistance outcome.

**Figure 10:**
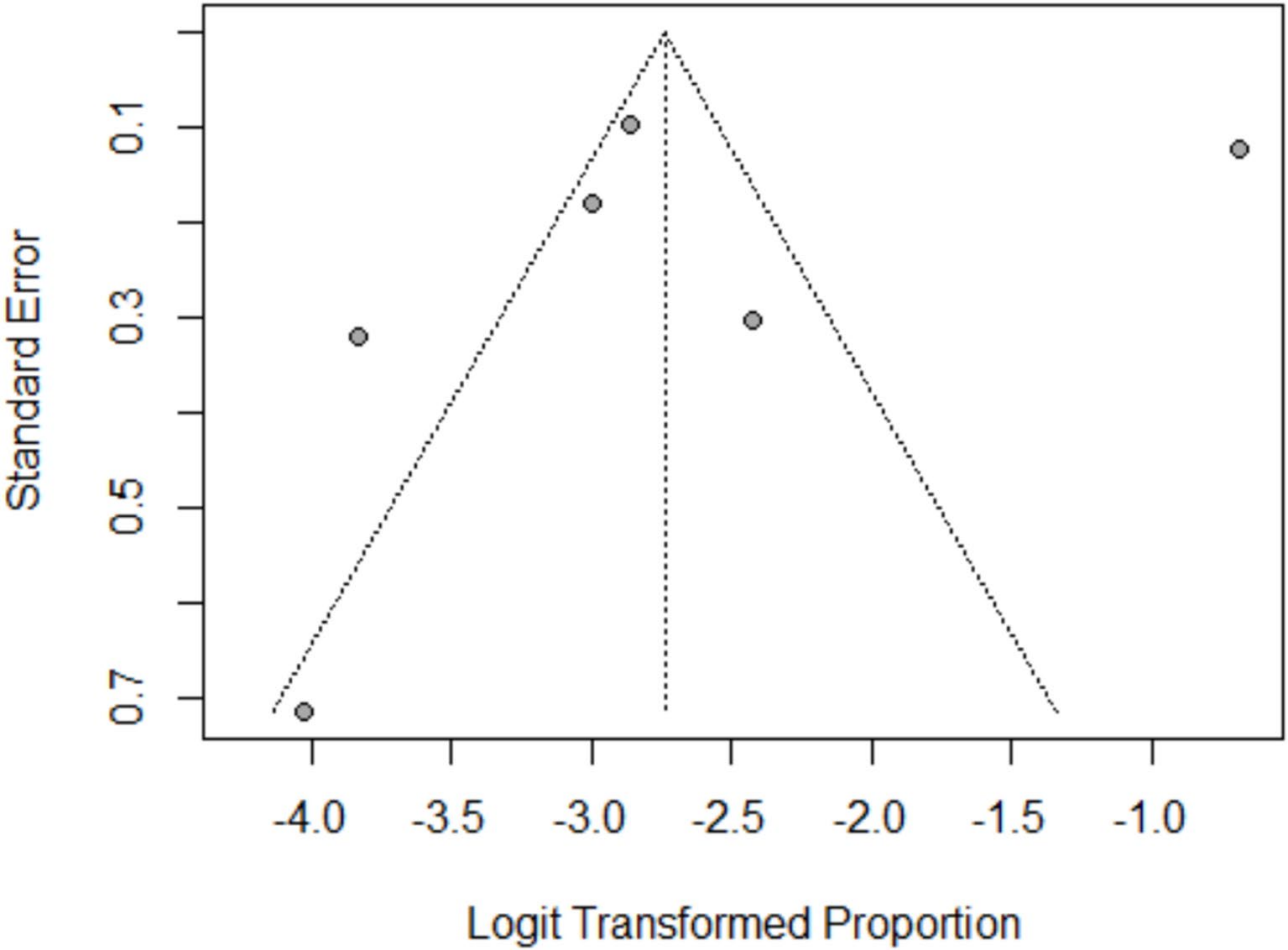
Funnel plot of the pyrazinamide-resistance outcome.

The pooled prevalence of pyrazinamide resistance was 0.06 with a 95% CI ranging from 0.02 to 0.14 (Figure 8). Sensitivity analysis revealed substantial heterogeneity among the included studies (*I*^2^ = 98.0%, *p* < 0.0001). The leave-one-out test did not resolve the heterogeneity after omitting Alshahrani et al. (12) (*I*^2^ = 71%) (Figure 9). The funnel plot for the pyrazinamide-resistance outcome is shown in Figure 10.

#### 3.3.4 Rifampicin resistance

**Figure 11:**
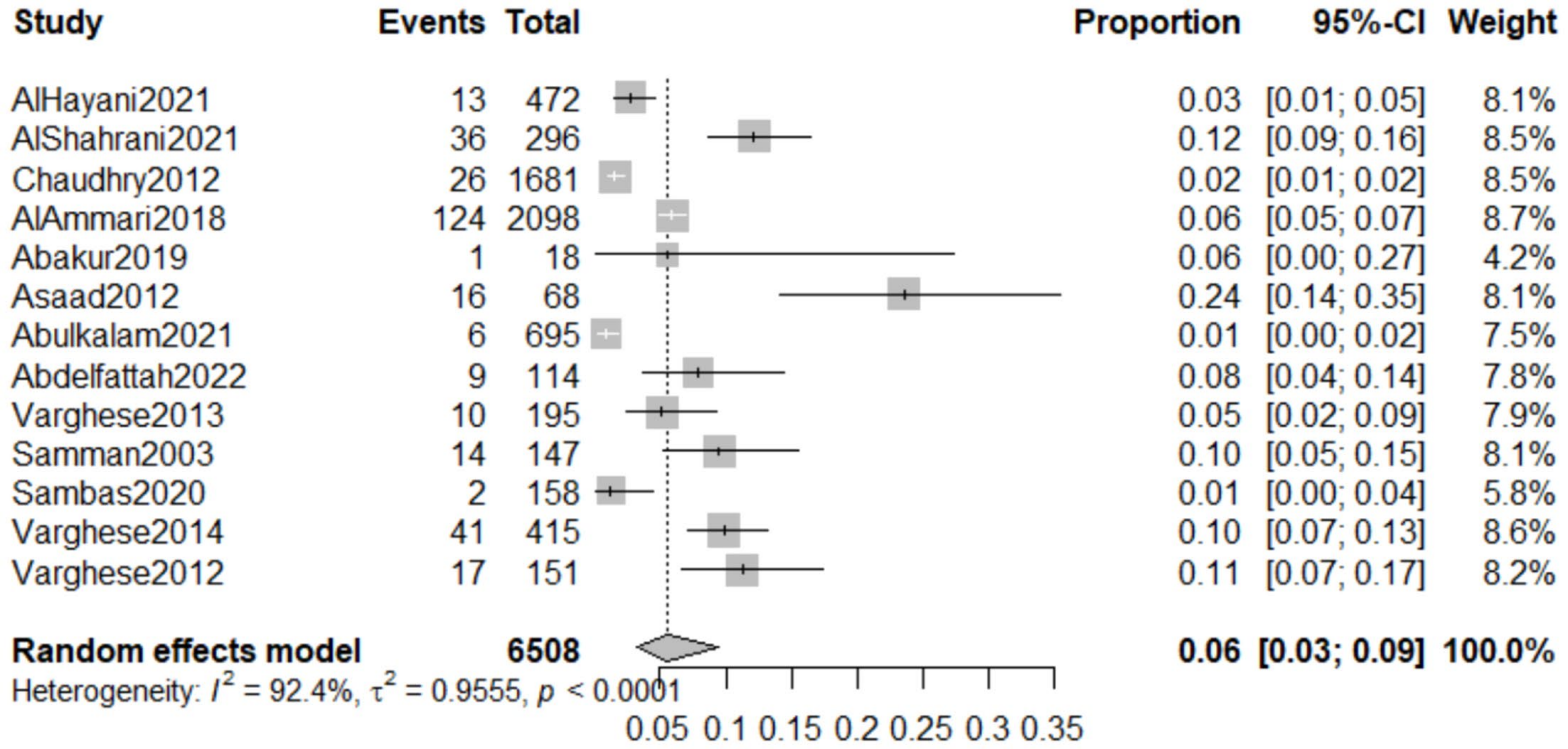
Forest plot of the rifampicin-resistance outcome.

**Figure 12:**
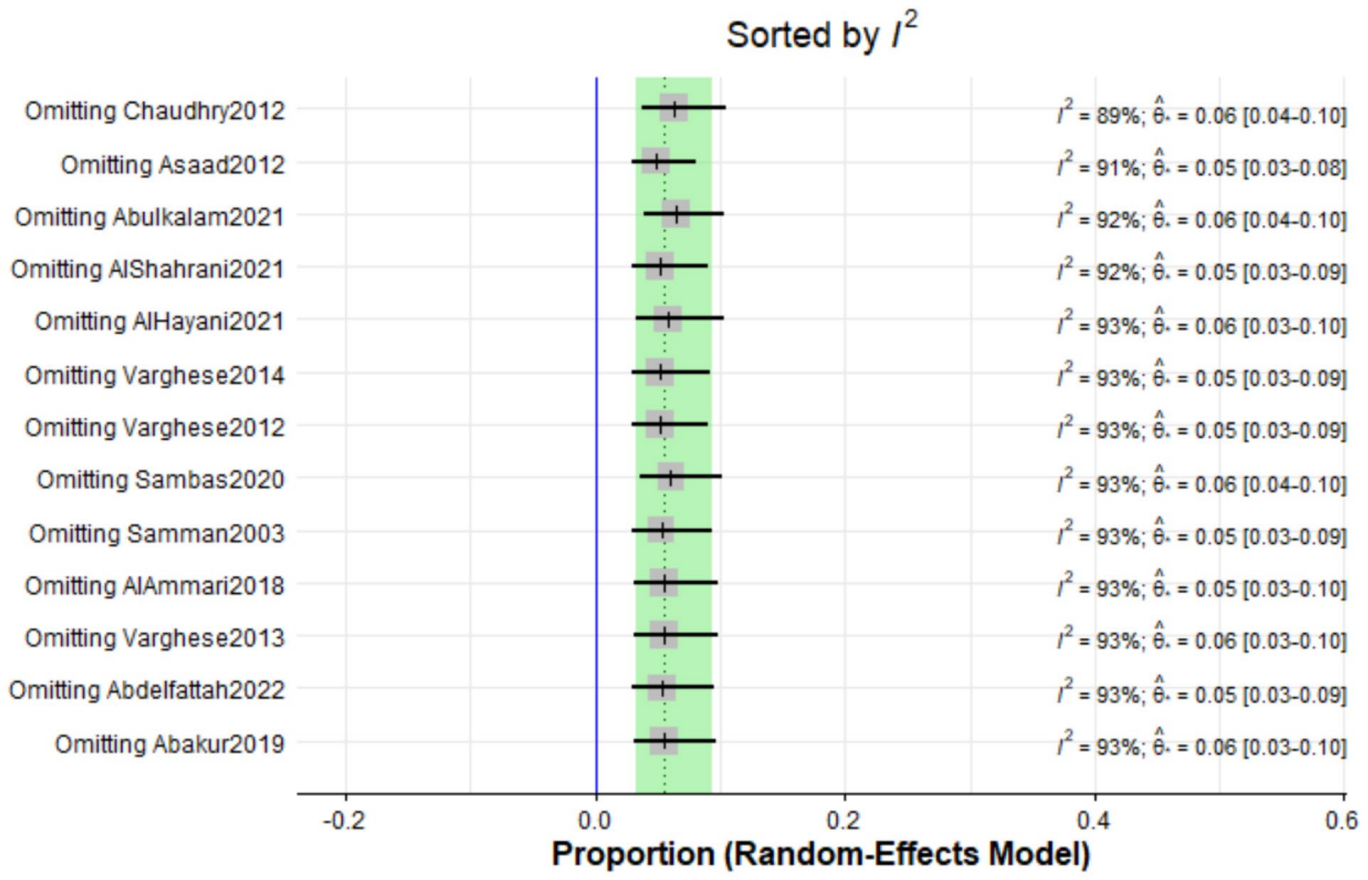
Sensitivity analysis of the rifampicin-resistance outcome.

**Figure 13:**
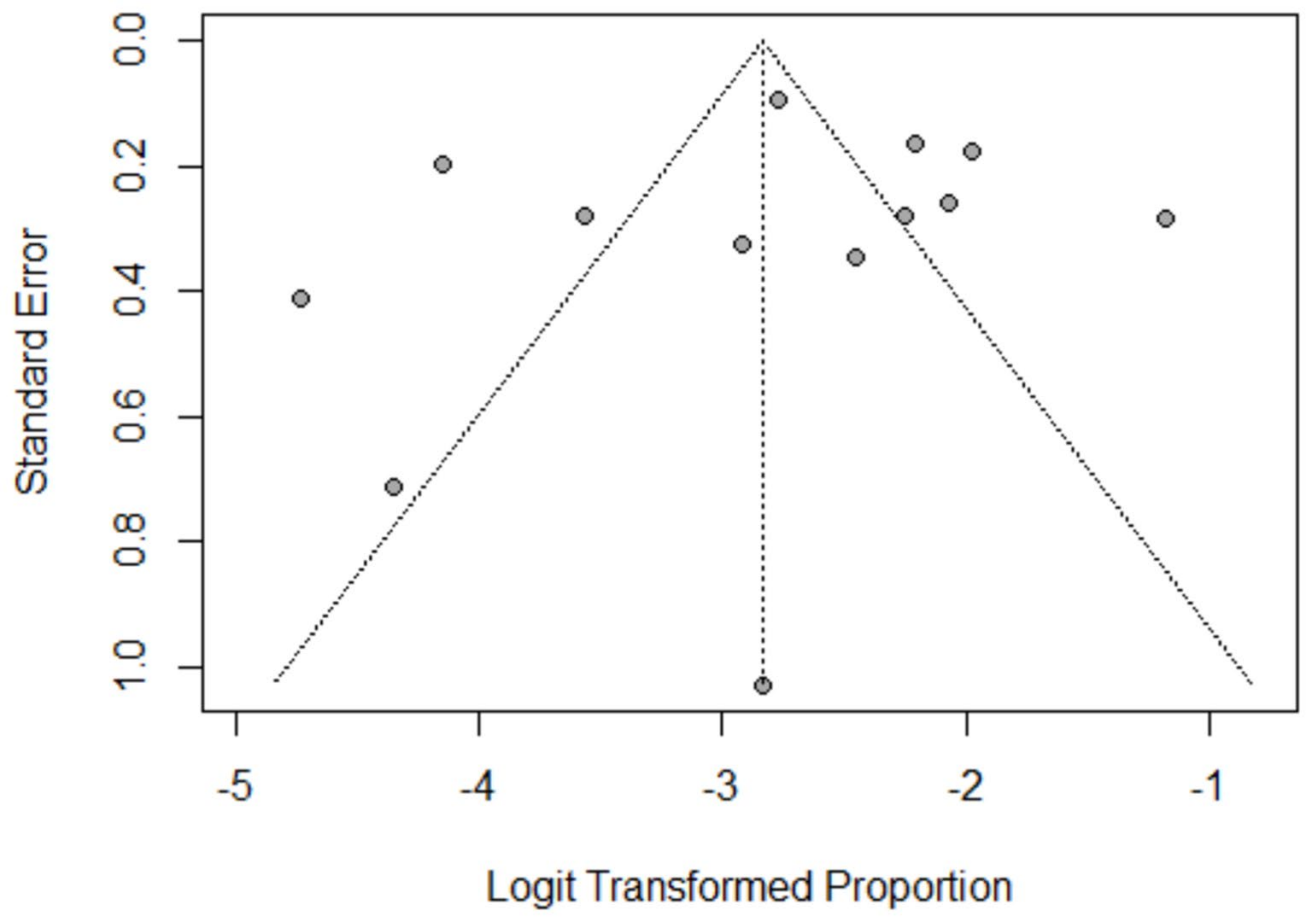
Funnel plot of the rifampicin-resistance outcome.

The pooled prevalence of rifampicin resistance was 0.06 with a 95% CI ranging from 0.03 to 0.09 (Figure 11). Sensitivity analysis revealed substantial heterogeneity among the included studies (*I*^2^ = 92.4%, *p* < 0.0001). The leave-one-out analysis did not resolve the heterogeneity after omitting Ali Chaudhry et al. (11) (*I*^2^ = 89%) (Figure 12). The funnel plot of rifampicin-resistance outcome is shown in Figure 13.

#### 3.3.5 Streptomycin resistance

**Figure 14:**
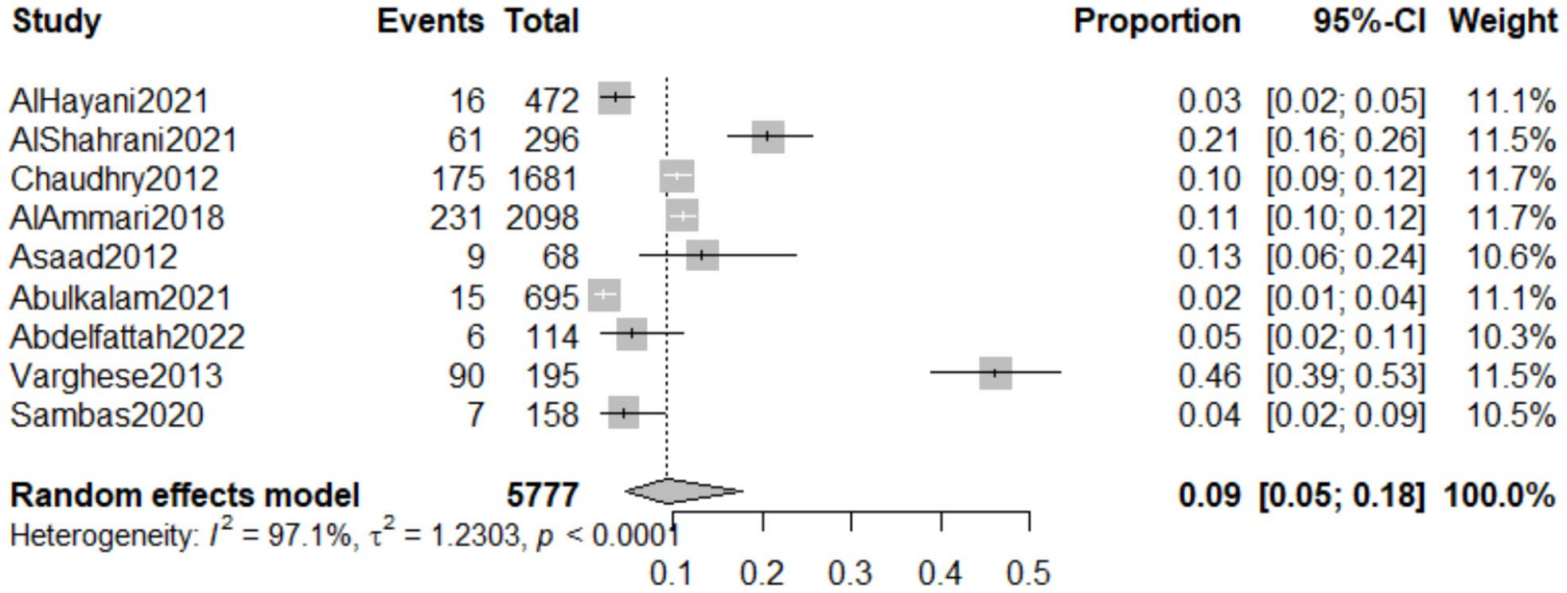
Forest plot of the streptomycin-resistance outcome.

**Figure 15:**
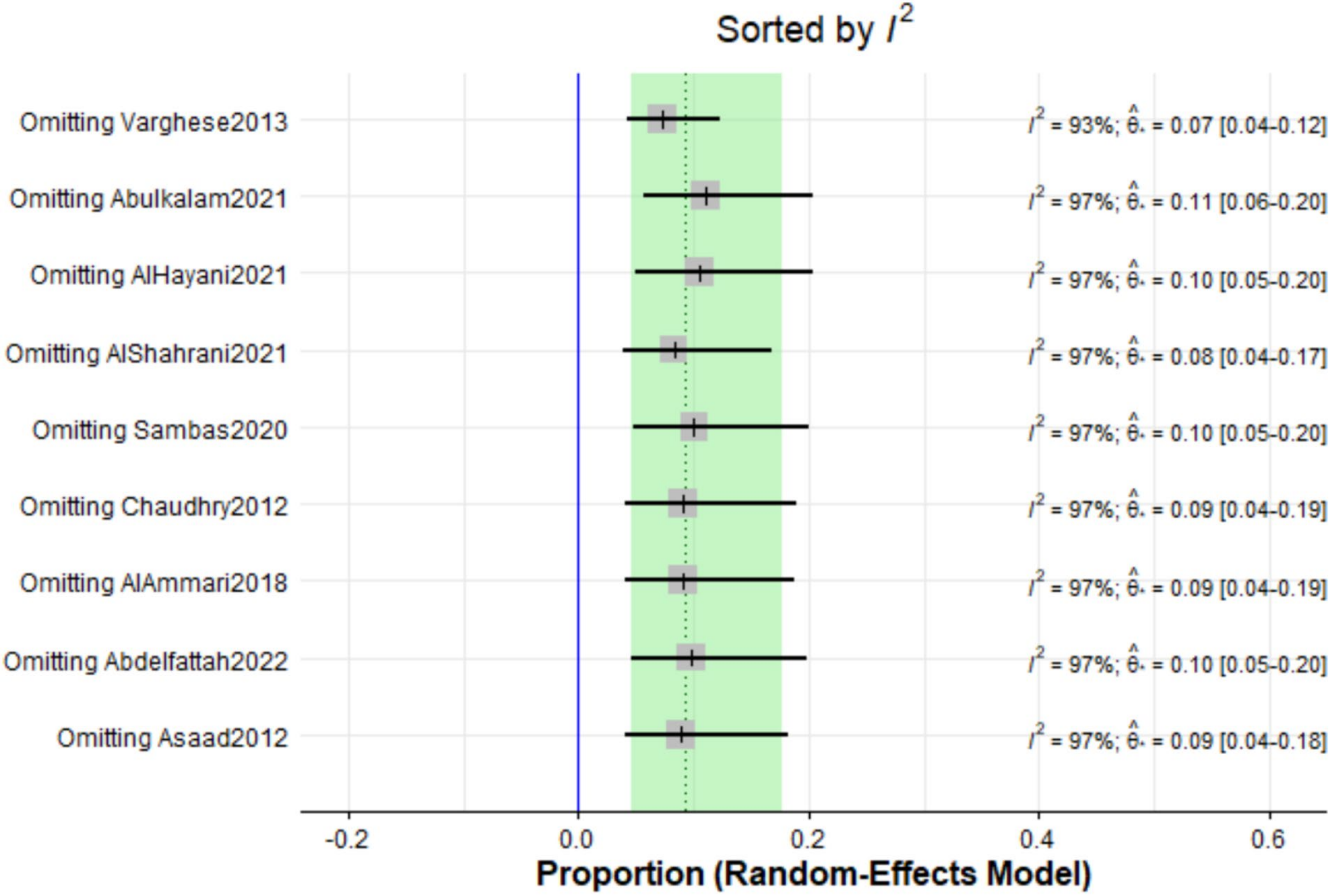
Sensitivity test of the streptomycin-resistance outcome.

**Figure 16:**
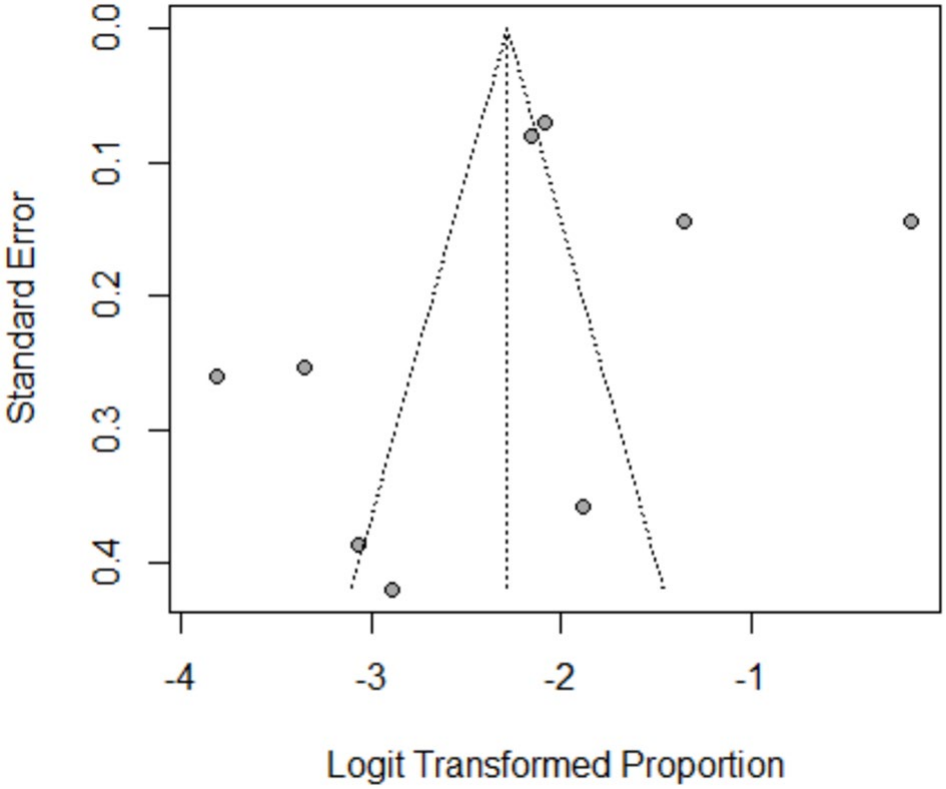
Funnel plot of the streptomycin-resistance outcome.

The pooled prevalence of streptomycin resistance was 0.09 with a 95% CI ranging from 0.05 to 0.18 (Figure 14). Sensitivity analysis revealed substantial heterogeneity among the included studies (*I*^2^ = 97.1%, *p* < 0.0001). The leave-one-out test did not resolve the heterogeneity after omitting Varghese et al. (13) (*I*^2^ = 93%) (Figure 15). The funnel plot for the streptomycin-resistance outcome is shown in Figure 16.

#### 3.3.6 Multidrug-resistant tuberculosis (MDR-TB)

**Figure 17:**
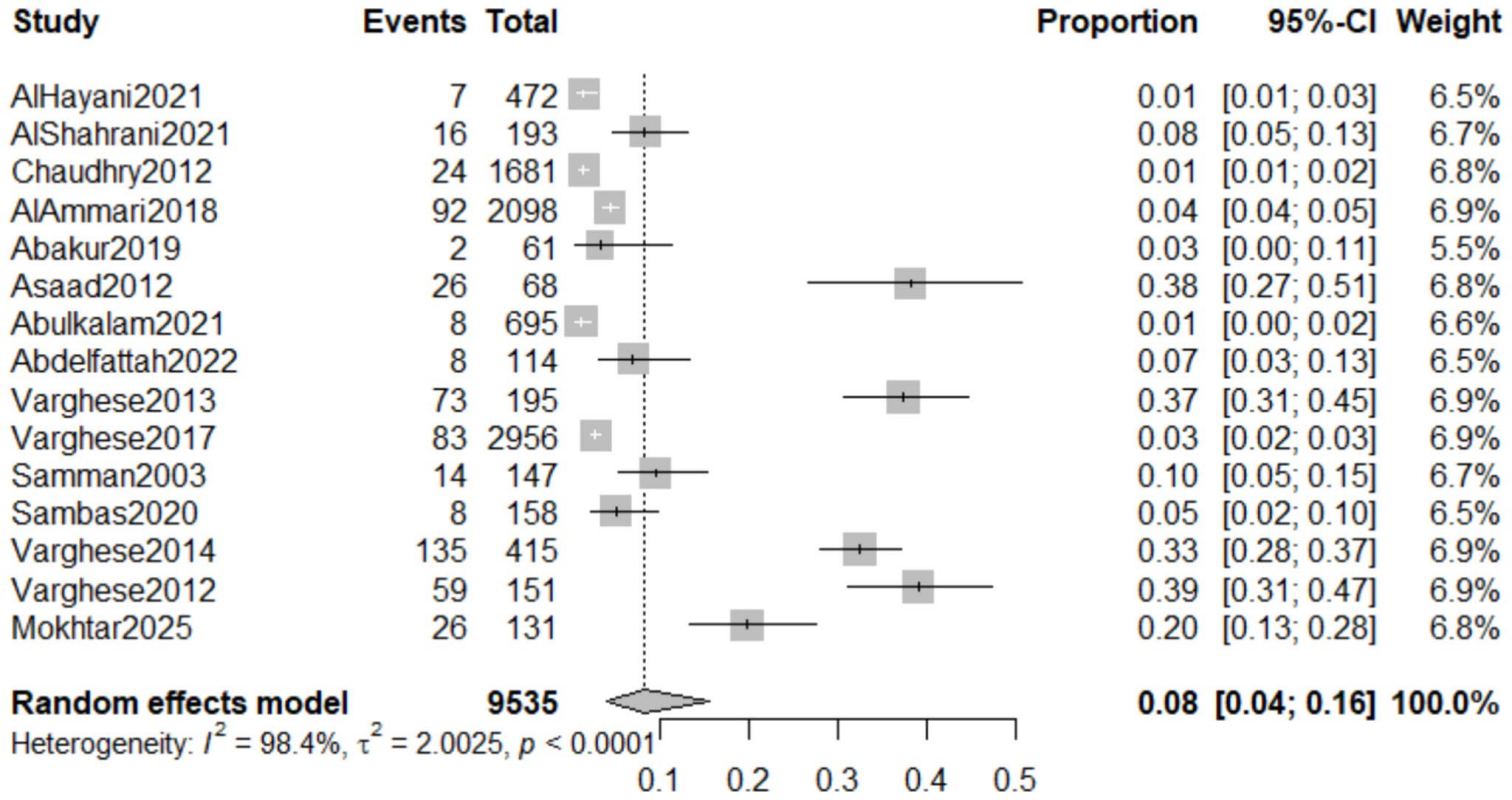
Forest plot of the pooled prevalence of multidrug-resistant tuberculosis (MDR-TB).

**Figure 18:**
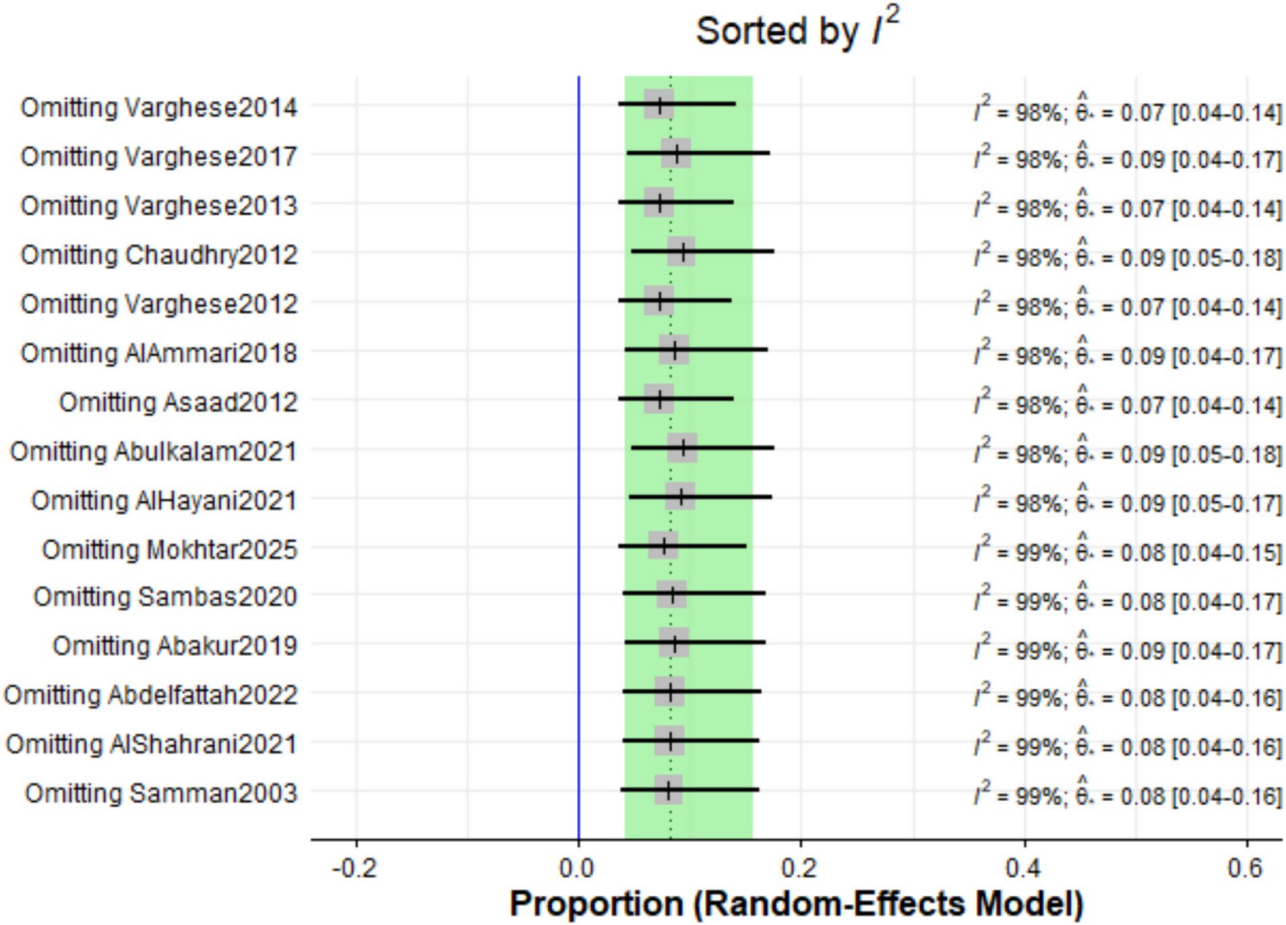
Leave-one-out sensitivity analysis of the pooled prevalence of multidrug-resistant tuberculosis (MDR-TB).

**Figure 19:**
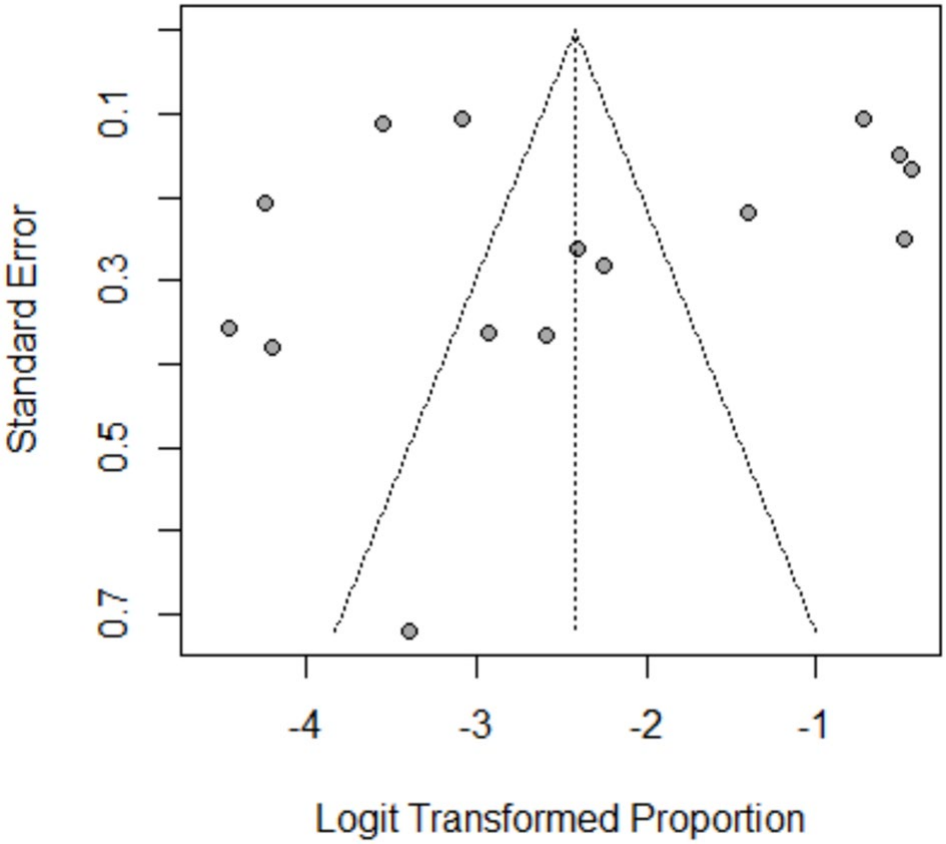
Funnel plot assessing small-study effects for the pooled prevalence of multidrug-resistant tuberculosis (MDR-TB).

The pooled prevalence of MDR-TB was 0.08 with a 95% CI ranging from 0.04 to 0.16 (Figure 17). Sensitivity analysis revealed substantial heterogeneity among the included studies (*I*^2^ = 98.4%, p < 0.0001). The leave-one-out test did not resolve the heterogeneity after omitting Varghese et al. (10) (*I*^2^ = 98%) (Figure 18). The funnel plot for the MDR-TB outcome is shown in Figure 19.

### 3.4 Secondary outcomes: risk factors associated with MDR-TB

#### 3.4.1 Gender risk factor

**Figure 20:**
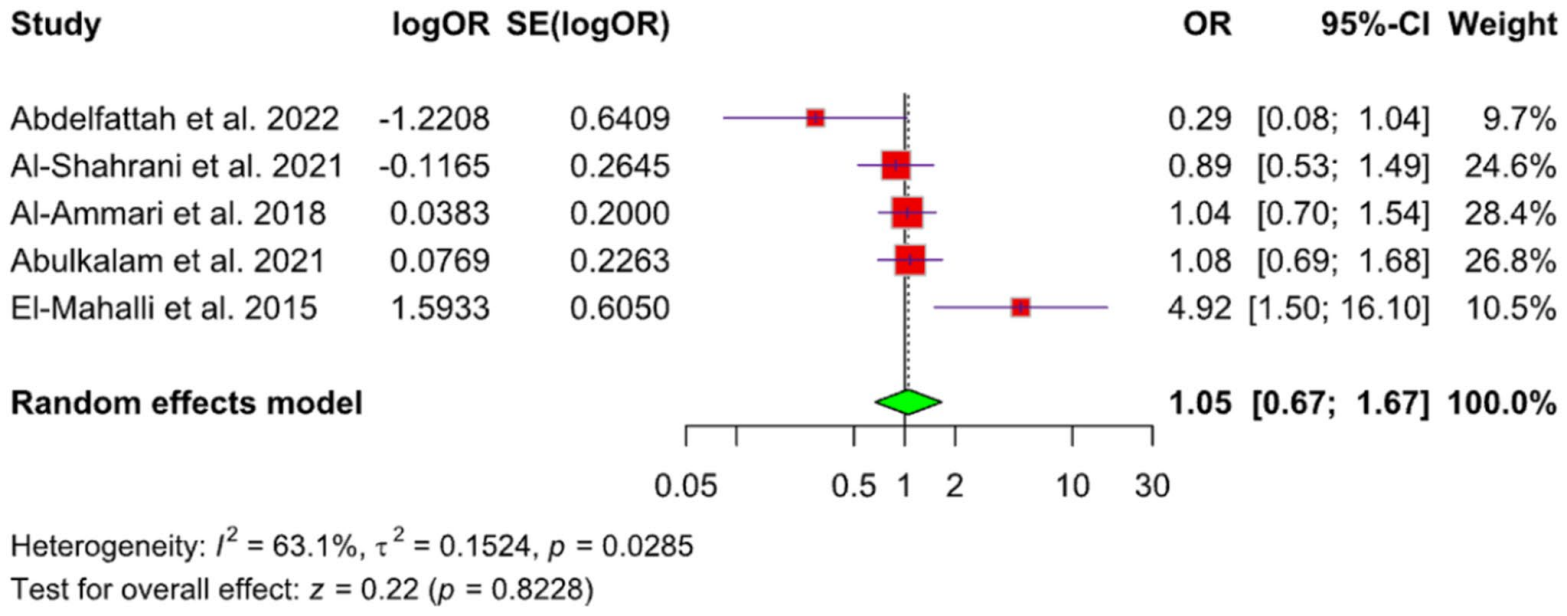
Forest plot of the association between male sex and multidrug-resistant tuberculosis (MDR-TB) using pooled unadjusted odds ratios.

**Figure 21:**
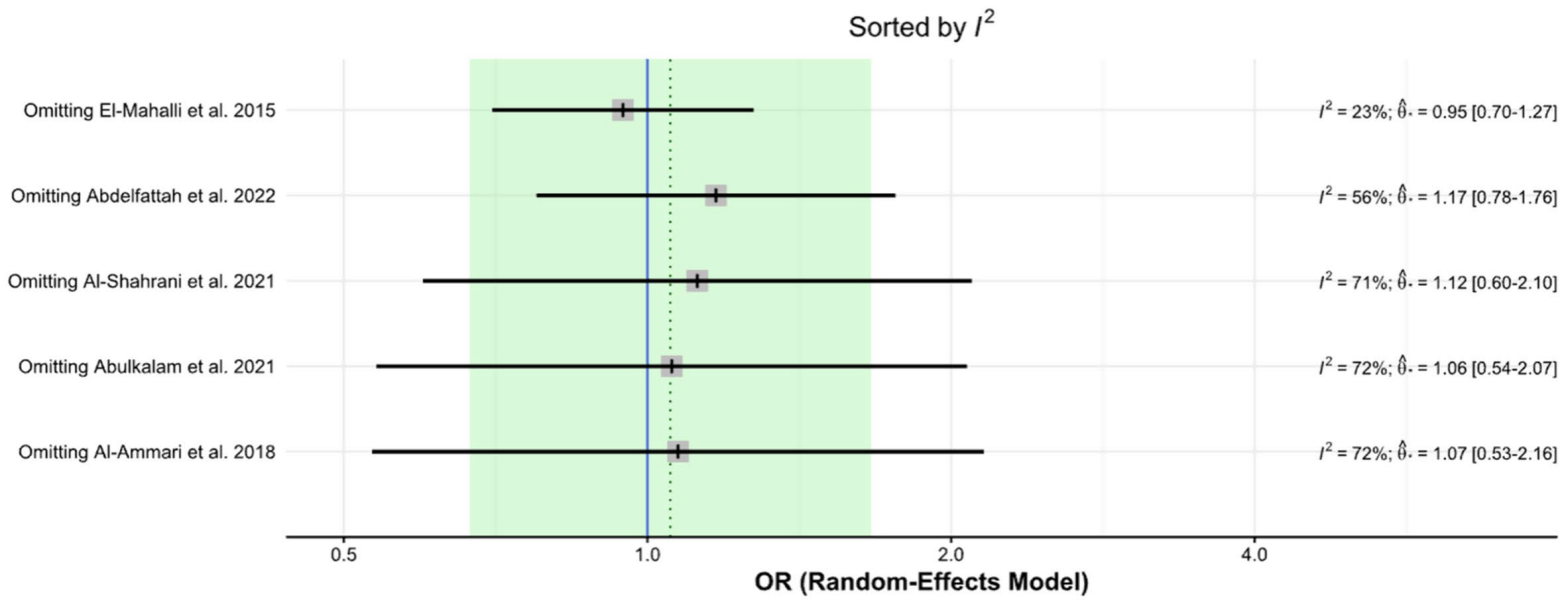
Leave-one-out sensitivity analysis of the association between male sex and multidrug-resistant tuberculosis (MDR-TB) using unadjusted odds ratios.

The pooled OR for male sex in MDR-TB was 1.05 with a 95% CI ranging from 0.67 to 1.67 across five studies (Figure 20). The result was not statistically significant (*p* = 0.8228). Sensitivity analysis revealed moderate heterogeneity among the included studies (*I*^2^ = 63.1%). The leave-one-out test resolved the heterogeneity after omitting El Mahalli and Al-Qahtani (14) (*I*^2^ = 23%) (Figure 21).

**Figure 22:**
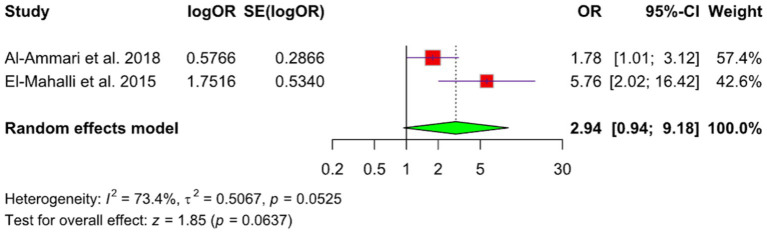
Forest plot of the association between male sex and multidrug-resistant tuberculosis (MDR-TB) using pooled adjusted odds ratios.

In contrast, the pooled adjusted OR of being male in MDR-TB resistance was 2.94 with a 95% CI ranging from 0.94 to 9.18 across two studies with a non-statistically significant result (*p* = 0.0637) (Figure 22).

#### 3.4.2 Nationality risk factor

**Figure 23:**
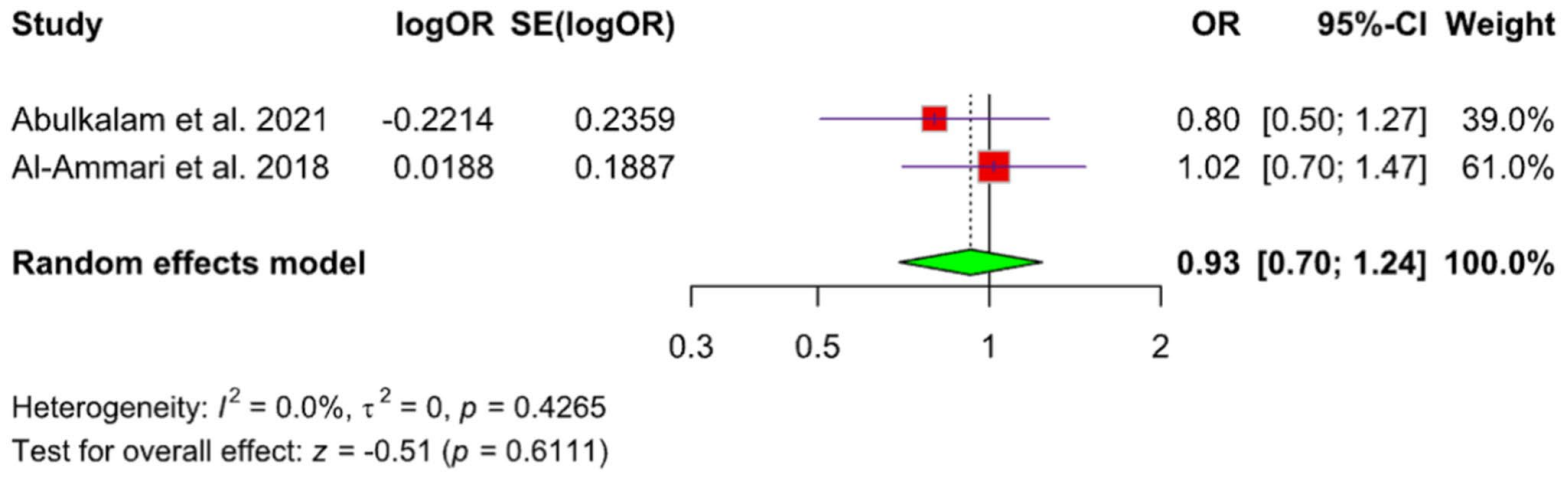
Forest plot of the association between Saudi nationality and multidrug-resistant tuberculosis (MDR-TB) using pooled unadjusted odds ratios.

**Figure 24:**
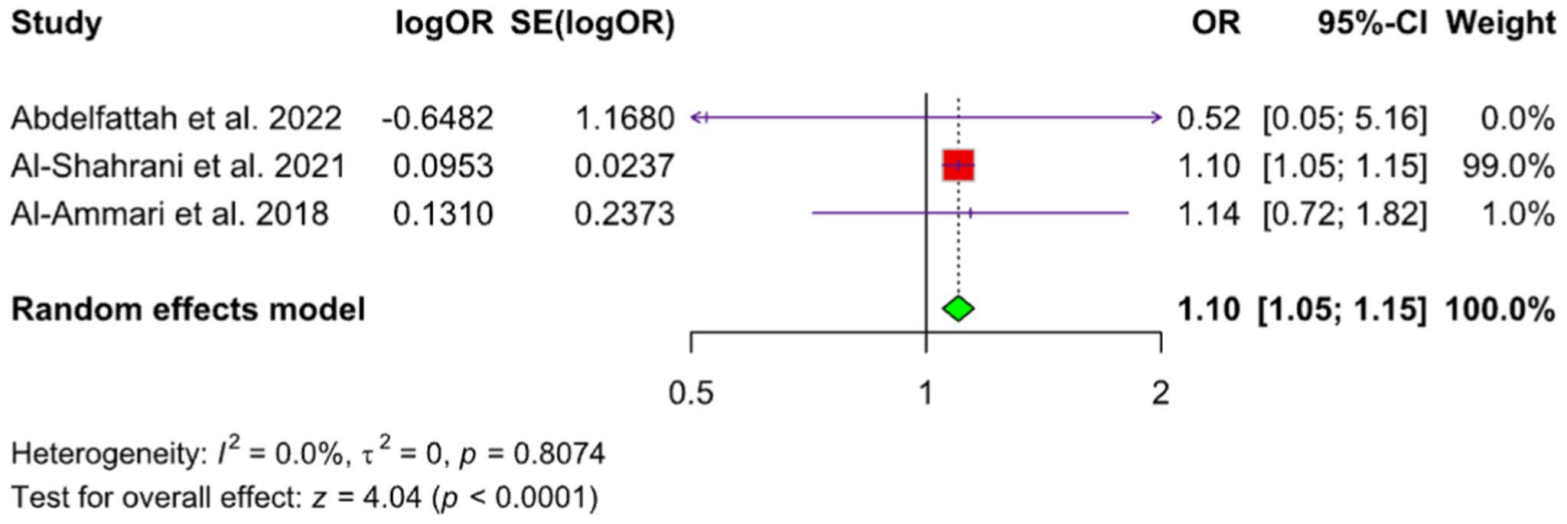
Forest plot of the association between Saudi nationality and multidrug-resistant tuberculosis (MDR-TB) using pooled adjusted odds ratios.

The pooled unadjusted OR for Saudi nationality and MDR-TB was 0.93, with a 95% CI ranging from 0.70 to 1.24 across two studies (Figure 23). This association was not statistically significant (*p* = 0.6111), with no observed heterogeneity (*I*^2^ = 0%). In contrast, the pooled adjusted OR for Saudi nationality and MDR-TB was 1.10, with a 95% CI ranging from 1.05 to 1.15 across three studies, indicating a statistically significant association (*p* < 0.0001), with no observed heterogeneity (*I*^2^ = 0%) (Figure 24).

#### 3.4.3 Smoking risk factor

**Figure 25:**
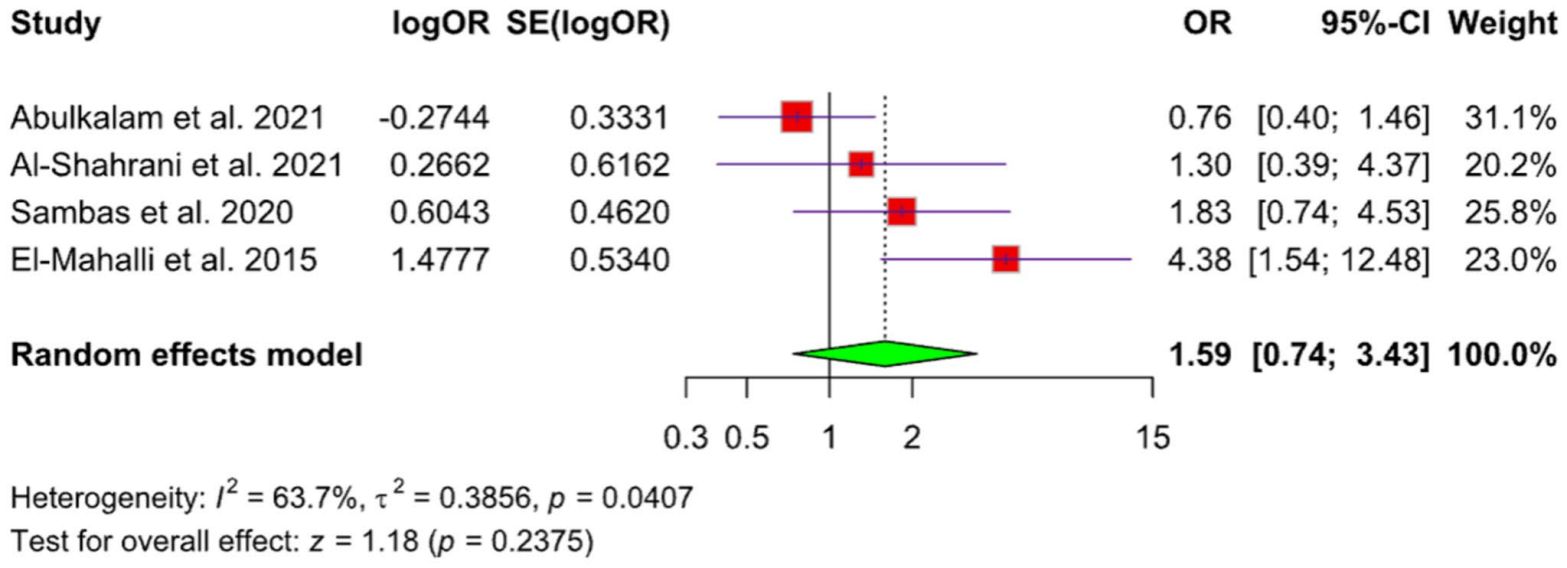
Forest plot of the association between smoking and multidrug-resistant tuberculosis (MDR-TB) using pooled unadjusted odds ratios.

**Figure 26:**
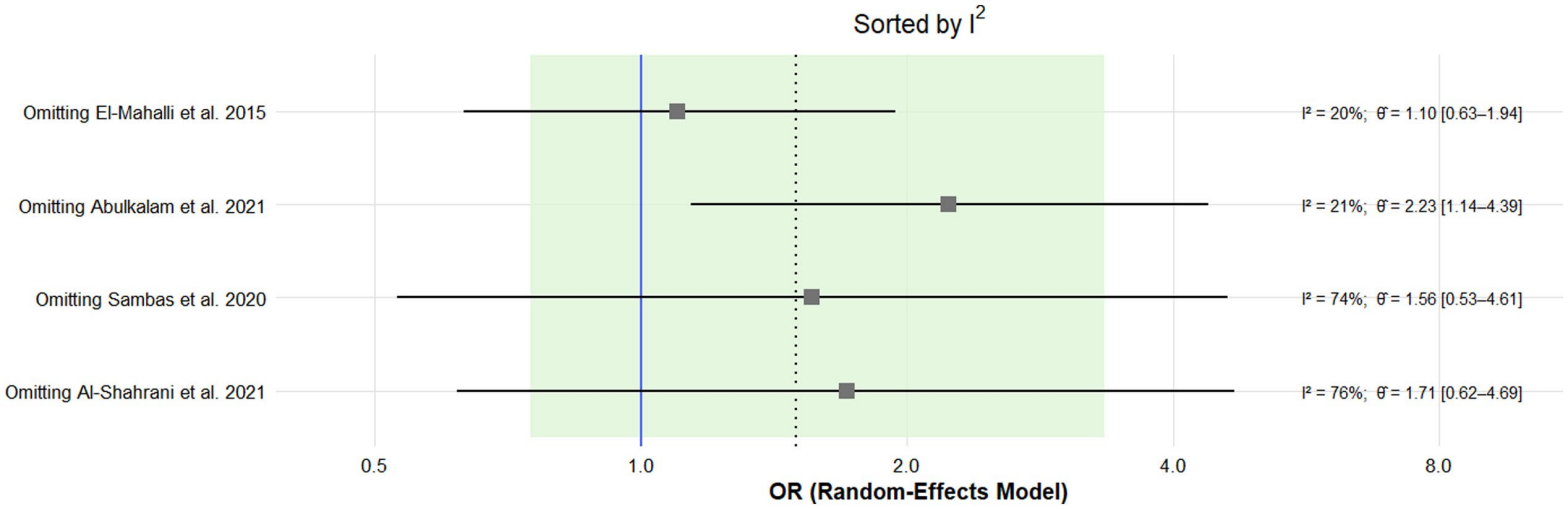
Leave-one-out sensitivity analysis of the association between smoking and multidrug-resistant tuberculosis (MDR-TB) using unadjusted odds ratios.

The pooled unadjusted OR for smoking and MDR-TB was 1.59, with a 95% CI ranging from 0.74 to 3.43 across four studies (Figure 25). This association was not statistically significant (*p* = 0.2375). Sensitivity analysis showed moderate heterogeneity among included studies (*I*^2^ = 63.7%), which was reduced after omitting El Mahalli and Al-Qahtani (14) (*I*^2^ = 20%) (Figure 26). In contrast, the pooled adjusted OR for smoking and MDR-TB was 3.23, with a 95% CI ranging from 1.53 to 6.84 across two studies, indicating a statistically significant association (*p* = 0.0021).

#### 3.4.4 Diabetes risk factor

**Figure 27:**
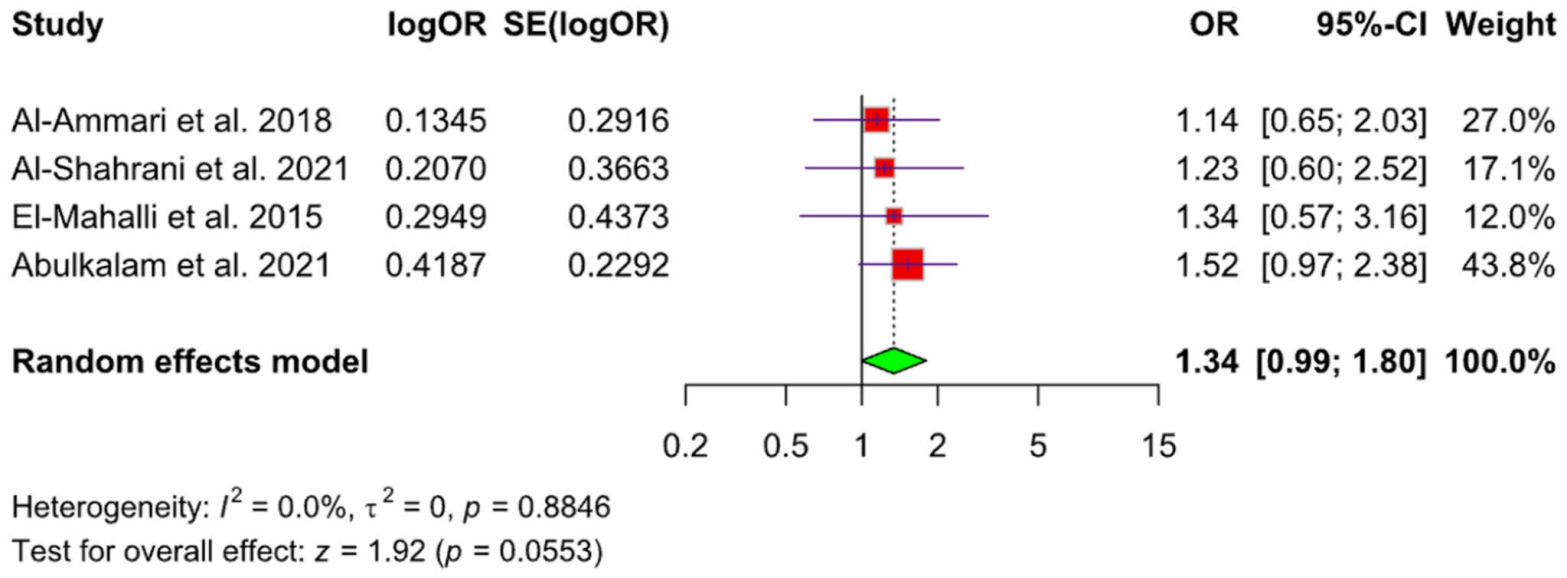
Forest plot of the association between diabetes mellitus and multidrug-resistant tuberculosis (MDR-TB) using pooled unadjusted odds ratios.

**Figure 28:**
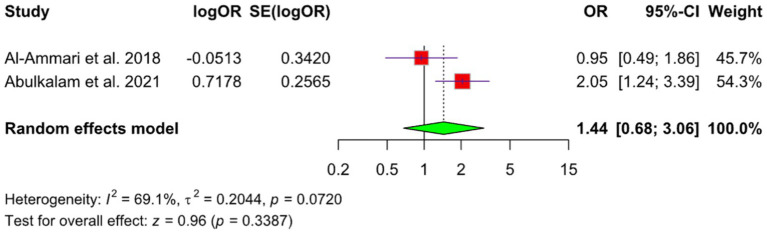
Forest plot of the association between diabetes mellitus and multidrug-resistant tuberculosis (MDR-TB) using pooled adjusted odds ratios.

The pooled odds ratio of diabetes in MDR-TB resistance was 1.34 with a 95% CI ranging from 0.99 to 1.80 across four studies (Figure 27). The result was not statistically significant (*p* = 0.0553). Sensitivity analysis revealed no heterogeneity among the included studies (*I*^2^ = 0%). In contrast, the pooled adjusted odds ratio of diabetes in MDR-TB was 1.44 with a 95% CI ranging from 0.68 to 3.06 across two studies with a non-statistically significant result (*p* = 0.3387) (Figure 28).

#### 3.4.5 Pulmonary site risk factor

**Figure 29:**
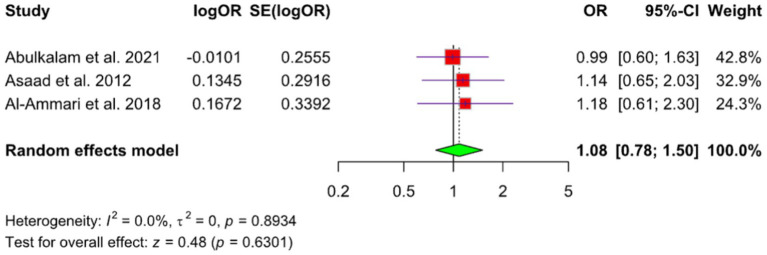
Forest plot of the association between pulmonary tuberculosis site and multidrug-resistant tuberculosis (MDR-TB) using pooled unadjusted odds ratios.

The pooled odds ratio for pulmonary TB site in MDR-TB was 1.08 with a 95% CI ranging from 0.78 to 1.50 across three studies (Figure 29). The result was not statistically significant (*p* = 0.6301). Sensitivity analysis revealed no heterogeneity among the included studies (*I*^2^ = 0%).

#### 3.4.6 Previous treatment risk factor

**Figure 30:**
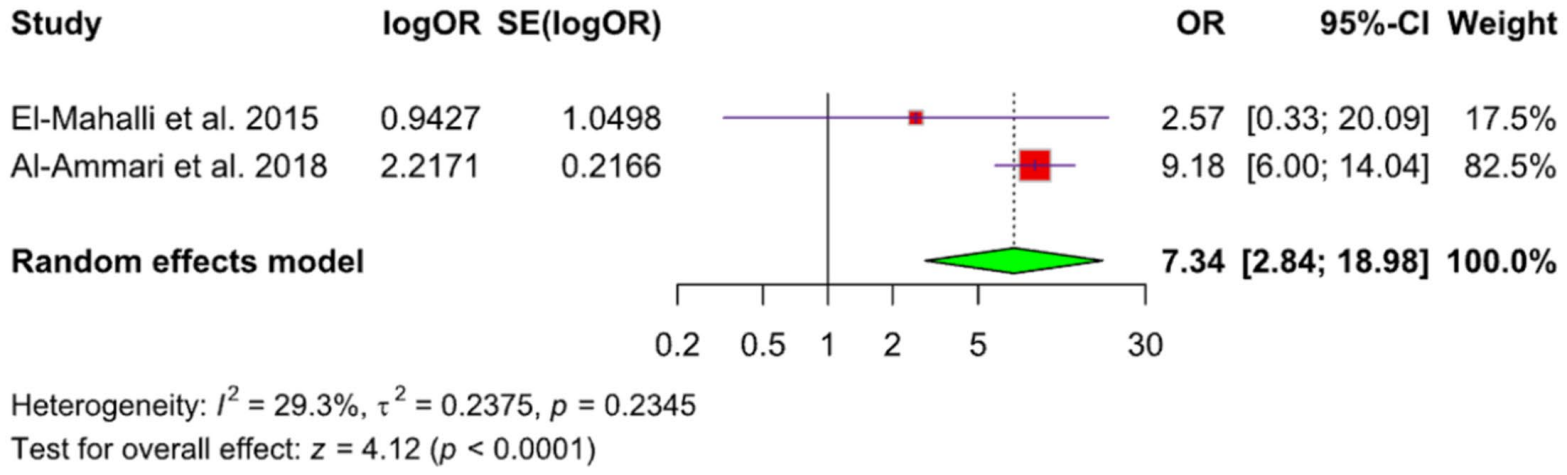
Forest plot of the association between previous TB treatment and multidrug-resistant tuberculosis (MDR-TB) using pooled unadjusted odds ratios.

The pooled odds ratio of previous TB treatment in MDR-TB was 7.34 with a 95% CI ranging from 2.84 to 18.98 across three studies (Figure 30). The result was statistically significant (*p* < 0.0001). Sensitivity analysis revealed no heterogeneity among the included studies (*I*^2^ = 29.3%).

## 4 Discussion

MDR-TB remains a significant challenge for healthcare systems worldwide, particularly in Saudi Arabia, due to its high treatment costs, side effects, and the complexity of disease management (15). Our study conducted a systematic review and meta-analysis to assess the prevalence of MDR-TB and identify associated risk factors such as age, smoking, TB history, comorbidities, and demographic characteristics. This combined approach allowed us to synthesize data from multiple quantitative observational studies conducted in Saudi Arabia from 2000 onward, as outlined in our study protocol.

The seemingly broad range of included studies reflects the limited number of Saudi studies specifically designed to investigate MDR-TB prevalence and associated risk factors. Thus, the synthesis was based on an outcome-based extraction method, whereby studies with broader aims were included if they reported extractable MDR-TB or first-line drug-resistance data. This method enabled the review to include relevant national evidence without pooling results unrelated to the predefined review question.

The pooled results from this systematic review and meta-analysis revealed a prevalence of 8% for MDR-TB in Saudi Arabia (95% CI: 0.04–0.16). This estimate highlights notable variability across the included studies, reflecting regional differences and inconsistencies in diagnostic methods. Nevertheless, the pooled prevalence provides an important indication of the MDR-TB burden in the country. Resistance rates were 15% for isoniazid, 6% for rifampicin, and 9% for streptomycin.

This resistance rate is higher compared with a study conducted in Saudi Arabia in 2015, which reported an MDR-TB prevalence of 6%, and other studies conducted in the Gulf Cooperation Council (GCC) region, where MDR-TB prevalence was reported at 4% in Saudi Arabia. Moreover, isoniazid was the most common form of monodrug resistance (16, 17).

In terms of risk factors, prior treatment with TB was most strongly and consistently associated with MDR-TB. In the unadjusted analyses, male sex, Saudi nationality, smoking, diabetes, and pulmonary TB site were not significantly linked with MDR-TB. However, adjusted pooled analyses indicated statistically significant associations for Saudi nationality and smoking, with smoking showing a strong association (adjusted OR = 3.23, 95% CI: 1.53–6.84, *p* = 0.0021). These adjusted results should be interpreted with caution because they were based on a limited number of studies and may have been influenced by variations in covariate adjustment, population structure, and residual confounding.

On the other hand, another meta-analysis found that non-Saudi nationality was associated with a 2–3-fold increased risk of developing MDR-TB (18). This variation in our findings may be explained by the country’s role as a major migration hub because of its unique geographical location at the crossroads of Africa, Asia, and Europe. In addition, the country annually hosts millions of Islamic pilgrims. Although approximately 60% are Saudi nationals, a substantial proportion arrive from regions with a high TB burden (19). Previous research has shown significant regional variation in tuberculosis drug-resistance patterns across the Kingdom, with a 2015 study in Saudi Arabia also reporting considerable interregional differences (16, 17).

Importantly, previous TB treatment was the strongest and most consistent predictor of MDR-TB, with an OR of 7.34, confirming its major role in increasing the risk of MDR-TB. These findings, while informative, may have been influenced by limitations such as small sample sizes, study heterogeneity, and unmeasured variables like healthcare access and regional TB control measures, which could explain some discrepancies in identifying significant risk factors.

These findings are consistent with previous studies. For example, global meta-analysis studies in 2024 found that previously treated TB cases were 5.427 times more likely to develop MDR-TB compared with cases with no previous TB therapy (18). Another study in Saudi Arabia reported that the prevalence of MDR-TB was low among new cases (1.8%) but higher among previously treated cases (15.9%) (19). A study in the GCC region also found statistically significant associations for previously treated cases receiving antituberculosis therapy (*p* = 0.001) (19).

Global meta-analysis studies in 2024 found no statistically significant associations for sociodemographic factors or comorbidities such as smoking, diabetes mellitus, human immunodeficiency virus (HIV) infection, and chronic obstructive pulmonary disease (COPD) (18).

In contrast, a study conducted in Saudi Arabia found that age and gender were significant risk factors for developing MDR-TB (6).

The heterogeneity observed in our systematic review and meta-analysis remains a key limitation. Variations in study designs, diagnostic methods, and patient demographics likely contributed to this heterogeneity. Different studies employed various diagnostic approaches, such as culture-based methods, drug-susceptibility testing, GeneXpert, and combined laboratory testing, which could explain differences in MDR-TB prevalence and the identification of risk factors. Additionally, some studies focused on specific populations, such as expatriates or particular regions of Saudi Arabia, which further contributed to the variability observed.

This high heterogeneity of several pooled prevalence outcomes is likely multifactorial. There was considerable variation in study design, setting, recruitment period, sample size, and population structure among the included studies. Some studies were hospital-based, whereas others relied on laboratory surveillance or regional data. Moreover, diagnostic methods differed across studies, ranging from traditional culture-based drug-susceptibility testing to molecular testing and combined laboratory approaches. This methodological variation may have contributed to differences in the reported prevalence of isoniazid resistance, rifampicin resistance, and MDR-TB. Between-study variability may also have been affected by differences in patient characteristics such as nationality, previous TB treatment, comorbidities, and regional distribution within Saudi Arabia.

### 4.1 Limitations and recommendations

This study has several limitations that should be considered when interpreting the results. The relatively small number of eligible studies and the small sample sizes in some studies limit both the statistical power and the generalizability of the findings. In addition, variations in study methodologies, diagnostic tools, and population characteristics introduced substantial heterogeneity, making it difficult to compare results across studies. Another important limitation was the inconsistent reporting of key risk factors, such as smoking, diabetes, and socioeconomic status, which prevented a comprehensive assessment of their association with MDR-TB. Moreover, the unadjusted and adjusted pooled estimates differed, especially for Saudi nationality and smoking, suggesting the potential influence of confounding and requiring cautious interpretation given the limited number of studies providing adjusted estimates. These factors, along with potential unmeasured variables such as healthcare access and regional TB control measures, may have influenced the discrepancies in risk factor identification.

To address these issues, future research in Saudi Arabia should focus on conducting large, well-designed cohort or case–control studies using standardized diagnostic methods and consistent reporting of patient-related factors. Expanding the diversity of study populations and settings beyond tertiary-care hospitals to include primary-care and rural areas, would also enhance the robustness and generalizability of the findings. Furthermore, future systematic reviews should ensure comprehensive data inclusion and harmonized definitions to reduce heterogeneity and provide more reliable evidence to guide TB control strategies.

## 5 Conclusion

This study highlights the ongoing challenge of MDR-TB and first-line anti-TB drug resistance in Saudi Arabia. The pooled MDR-TB prevalence was higher than several previous regional estimates, and previous TB treatment emerged as the most consistent predictor of MDR-TB. Adjusted analyses also suggested potential associations between smoking and Saudi nationality and MDR-TB; however, these findings should be interpreted with caution because of the limited number of contributing studies. These findings call upon physicians, program managers, and policymakers to prioritize interventions that support patients, ensure treatment adherence, and strengthen TB control programs. Future research should focus on large, well-designed cohort studies with consistent reporting of patient-related factors, expanded populations, and diverse settings. Moreover, systematic reviews in the future should ensure comprehensive data inclusion and harmonized definitions to minimize heterogeneity and provide more reliable evidence to guide TB control strategies. In conclusion, addressing MDR-TB requires robust research, stronger surveillance, and tailored interventions to reduce its burden in Saudi Arabia.

## Data Availability

The original contributions presented in the study are included in the article, further inquiries can be directed to the corresponding author.
